# Regionally divergent drivers behind transgressions of the freshwater change planetary boundary

**DOI:** 10.1038/s41467-026-73051-x

**Published:** 2026-05-13

**Authors:** Vili Virkki, Lauren Seaby Andersen, Sofie te Wierik, Dieter Gerten, Miina Porkka

**Affiliations:** 1 Department of Environmental and Biological Sciences, University of Eastern Finland, Joensuu, Finland; 2 Potsdam Institute for Climate Impact Research, Member of the Leibniz Association, Potsdam, Germany; 3 Netherlands Environmental Assessment Agency (PBL), The Hague, The Netherlands; 4 Geography Department, Humboldt-Universität zu Berlin, Berlin, Germany; 5 Integrative Research Institute on Transformations of Human-Environment Systems, Humboldt-Universität zu Berlin, Berlin, Germany

**Keywords:** Hydrology, Hydrology

## Abstract

Human-driven freshwater change relates to elevated Earth system risks, which motivates analysis to better understand its global characteristics. Here, we analyse global and regional patterns of anomalous conditions and their drivers in streamflow (blue water) and soil moisture (green water), building on the recently updated planetary boundary for freshwater change (PB-FW). Our data consist of updated scenario simulations from a large ensemble of global hydrological models covering years 1901–2019. During the early twenty-first century, PB-FW transgression has increased across its blue and green water components. Climate has increasingly become the dominant global influence on dry and wet streamflow and soil moisture deviations from pre-industrial-like baseline conditions. Amongst the regionally variable change in blue and green water, direct human forcings (encompassing land and water use changes) intensify particularly dry deviations, whereas wet deviations are mainly climate-driven. Regional unpacking of the global PB-FW transgression improves understanding of the extent, degree and drivers of global freshwater change, guiding mitigation and adaptation strategies in response to it, and is a notable advancement for analysing PBs across scales.

## Abstract (web summary)

The study shows how human pressures are increasingly pushing freshwater systems beyond the planetary boundary for freshwater change. Climate change is the dominant global driver, while water and land use intensify particularly dry streamflow and soil moisture anomalies across many regions.

## Introduction

Freshwater plays an integral role in the functioning of the Earth system^1^, stability of which has allowed for the development of current societies^2^. Human activities, however, continue to modify the freshwater cycle, by direct water withdrawals and flow regulation and through anthropogenic climate and land cover change, for instance^3–7^. As freshwater is deeply connected to terrestrial and aquatic ecological processes, anomalous hydrological conditions—including increased frequency, magnitude and variability of drying or wetting across water cycle elements—can destabilise ecosystems where freshwater availability governs their resilience^8–10^. Moreover, freshwater change modifies climatic processes by, for instance, altering land-atmosphere carbon exchange^11,12^ and moisture recycling^13^, often in a teleconnected manner that propagates impacts beyond their origin and across scales^14^. Although thresholds for adverse impacts of freshwater change can be case-specific and uncertain^15^, deviations from historically stable hydrological conditions are likely associated with increasing risks of both abrupt and gradual ecological and climatic impacts^16,17^. Cumulatively with other human-driven impacts on the Earth system, disruptions of the freshwater cycle thus contribute to a feedback loop; while societies depend on a stable Earth system, they create pressures that threaten its continued stability and then put themselves at an increasing risk. This interplay motivates a strong need to assess and understand freshwater change at the global scale.

The planetary boundaries (PBs) framework aims for understanding and assessing multiple anthropogenic perturbations to the Earth system and the risks they pose to vitally important Earth system processes^18^. The recently updated PB for freshwater change (PB-FW) builds on the premise that increasingly frequent anomalous hydrological conditions serve as indicators of broader stress on freshwater’s Earth system functions^16,17^. The PB-FW is shown to be notably transgressed, which marks an elevated level of Earth system risks due to freshwater change^18^, aligning with evidence on severe freshwater-related adverse impacts on societies^19,20^. Unlike earlier approaches based on global human water consumption, calculated top-down based on global consumption^2^ or bottom-up based on regional appropriation of rivers’ environmental flow requirements^21,22^, the new PB-FW integrates the evolution of both blue water (streamflow) and green water (soil moisture) changes in a consistent assessment scheme. It allows for spatial disaggregation of the PB-FW, offering a globally coherent evaluation considering regional patterns of change, and thus a valuable conceptual foundation for understanding human-driven freshwater change.

The recent PB-FW analysis by Porkka et al.^16^ identified broad change patterns across global, regional and local scales but did not quantitatively explore historical drivers of the PB-FW transgression trajectory. Moreover, the analysis was limited by its data spanning only until 2005, therefore missing more recent global hydrological change. It is important to consider those gaps because drivers and impacts of freshwater change vary widely across space and time^7,23–27^, and recent decades have shown intense change in the freshwater cycle^3,28–30^. Addressing these gaps using the newly proposed PB-FW methodology, in particular, is essential to link the global PB-FW metrics to regional drivers of freshwater change, as those drivers ultimately cause the hydrologic anomalies that underlie the globally aggregated PB-FW transgression. Having this knowledge, the PB-FW and the PBs framework can better be utilised for mitigating or adapting to Earth system risks related to freshwater change^31,32^.

In this study, we isolate major historical drivers of the global PB-FW transgression trajectory and analyse their regional decomposition. First, we reassess the currently available state-of-the-art PB-FW analysis and extend it to the year 2019, adding 15 years to the previous estimate^16^, using a large ensemble of updated hydrological model simulations. We then decompose the historical contributions of direct human forcing (DHF; including water use and land cover change, for instance) and climate-related forcing (CRF) to PB-FW metrics at global and regional scales, which advances from the previous PB-FW analysis by providing an in-depth outlook into the global and regional drivers of freshwater change. This demonstrates how the PB-FW can be used as a tool to study typologies of regions and configurations of freshwater change, which may contribute to freshwater-related risks in the Earth system, given historical dependencies and impacts on blue and green water resources.

## Results

The methodological framework of this study remains consistent with the newest PB-FW definition as proposed by Porkka et al.^16^. We first established local variability bounds for both streamflow and soil moisture at the grid cell scale (between local 5th–95th percentile values) under a baseline scenario that is largely undisturbed by human actions (“Methods”). Subsequently, we determined local deviations—i.e. events of anomalous dry and wet streamflow or soil moisture conditions falling outside of the 5th–95th percentile range—and estimated the occurrence of these deviations by aggregating them to the percentage of global or regional land area with local deviations (“Methods”). Variability in the occurrence of local deviations under the baseline scenario was finally used to set two reference boundaries at the global scale and for each region: one at the median of baseline variability and one at the upper end (95th percentile) of baseline variability (“Methods”). Following Porkka et al.^16^, we assume that widespread deviations beyond the baseline scenario variability elevate gradual or abrupt systemic risks related to freshwater change and thus use its upper end as a reference boundary to represent these risks. At the global scale, the upper end reference boundary corresponds to the PB-FW^16,18^.

We employed a large ensemble of state-of-the-art global hydrological model (GHM) outputs from the Inter-Sectoral Impact Model Intercomparison Project (ISIMIP) simulation round 3a^33,34^. The data, covering years 1901–2019, are simulated using reanalysis-based CRF together with dynamic DHF. To establish the baseline scenario, we used counterfactual simulations that assumed fixed DHF at 1901 levels and, respectively, CRF derived from detrended historical climate reanalysis data (“Methods”). We attributed hydrological changes to CRF and DHF by a scenario comparison, which is a generic attribution approach that has commonly been used in global water cycle change research^3,26^. In this approach, the historical scenario and hypothetical scenarios with only CRF or DHF applied were compared to the counterfactual baseline scenario; differences to the baseline scenario were statistically tested for significance and defined as scenario contributions (“Methods”).

### Historical CRF and DHF contributions on PB-FW

**Fig. 1:**
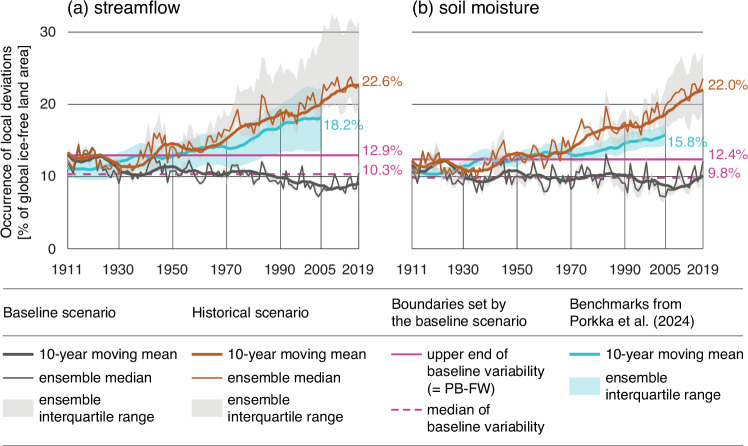
Global occurrence of local deviations. The global occurrence of local deviations is measured by the percentage share of global ice-free land area with dry and wet local deviations, for streamflow (**a**) and soil moisture (**b**). Shown is the ensemble median of annual percentage, which is computed as an average of monthly percentages (“Methods”). The occurrence of local deviations in 2019 (mean of 2010–2019) marks the current status of the planetary boundary for freshwater change (PB-FW). The baseline and historical scenario ensemble median and ensemble interquartile range are computed from *n* = 12 (streamflow) and *n* = 4 (soil moisture) ensemble members (”Methods”). Benchmark values until year 2005 are taken from the previous PB-FW status estimate by Porkka et al.^16^.

The degree of PB-FW transgression has increased for both streamflow and soil moisture during the late twentieth and early twenty-first century (Fig. 1). Local deviations during the last decade of our analysis (mean of 2010–2019) affected 22.6% of the global ice-free land area for streamflow (~29.4 million km^2^) and 22.0% for soil moisture (~28.6 million km^2^). These figures are 9.7 percentage points (pp) (75%) and 9.6 pp (77%) higher than the upper end of baseline variability that represents the PB-FW and is 12.9% and 12.4% for streamflow and soil moisture, respectively (Fig. 1).

The global occurrence of local deviations transgressed the PB-FW at around the 1940s (Fig. 1), which demonstrates long-standing alterations in the water cycle. After this, the freshwater cycle has increasingly deviated beyond the baseline conditions with little sign of stabilisation. During 1990–2019, the global land area affected by local streamflow and soil moisture deviations increased with an average rate of 0.19 pp/year and 0.23 pp/year, respectively (linear Theil-Sen regression slope; statistically significant by two-sided Kendall test^35^ at significance level *p* = 0.05). Notwithstanding the PB-FW transgression beginning already in the 1940s, more than half (5.7–6.9 pp) of the total transgression in 2019 has thus occurred during the late twentieth and early twenty-first century.

Differences in hydrological model ensembles (“Methods”) are apparent in how our estimates differ from the previous PB-FW status estimate by Porkka et al.^16^. Our current reassessment suggests that the previous PB-FW estimate may have been conservative; at the endpoint of the previous estimate in 2005, our new estimate is 2–3 pp higher compared to the previous estimate (18.2% and 15.8%; Fig. 1). Relative to the PB at the upper end of baseline variability, however, differences between the two PB-FW transgression estimates appear not equally large. This is because the reassessment places the PB-FW at 12.4–12.9% (Fig. 1), whereas the previous estimate^16^ placed it around 10–11%.

**Fig. 2:**
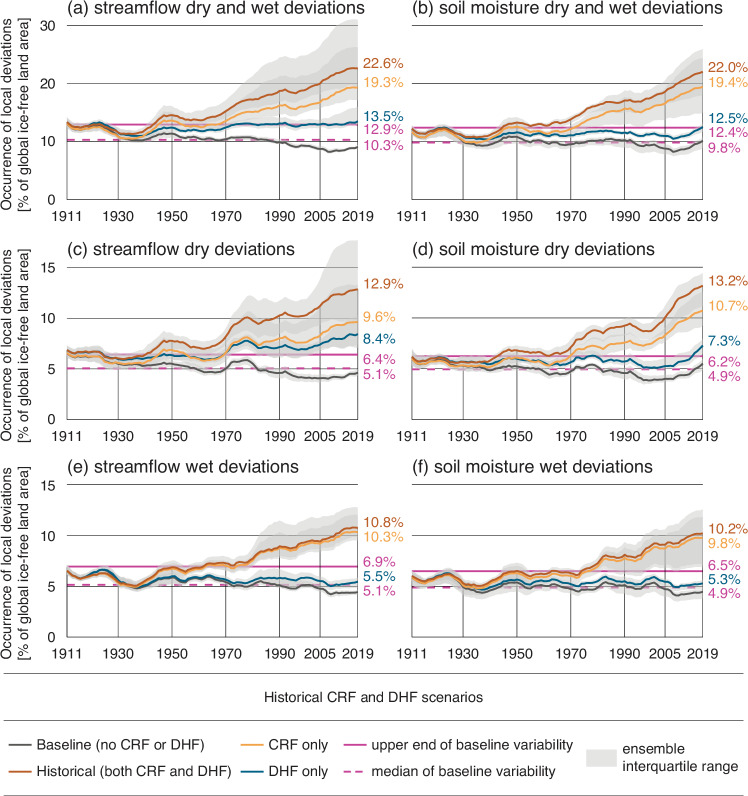
Global occurrence of dry and wet local deviations under different climate-related forcing (CRF) and direct human forcing (DHF) scenarios. The global occurrence of local deviations is measured by the percentage share of global ice-free land area with local deviations, for dry and wet streamflow deviations (**a**), dry and wet soil moisture deviations (**b**), dry streamflow deviations (**c**), dry soil moisture deviations (**d**), wet streamflow deviations (**e**), and wet soil moisture deviations (**f**). The baseline and historical scenarios in **a** and **b** correspond to Fig. 1a and Fig. 1b, respectively. Shown is the ensemble median of annual percentage, which is computed as an average of monthly percentages (“Methods”). Time series of the occurrence of local deviations and limits of the ensemble interquartile range (IQR) are smoothed with a 10-year moving (trailing) mean over the ensemble median and ensemble IQR limits, respectively.

Dry deviations are more widespread compared to wet deviations for both streamflow and soil moisture (Fig. 2c–f). However, local deviation occurrence in all four subcomponents of the PB-FW (i.e. dry and wet deviations, streamflow and soil moisture) show more than a doubling compared to the median of baseline variability that depicts a typical deviation occurrence value under the baseline scenario (Fig. 2c–f). In the case of dry deviations, local deviation occurrence shows more than a doubling even when compared to the upper end of baseline variability (Fig. 2c, d). Between 1990–2019, the occurrence of dry streamflow and soil moisture deviations increased at the fastest rates (0.13 pp/year; 0.23 pp/year, respectively) (Fig. 2c, d), while wet streamflow and soil moisture deviations increased at more gradual rates (0.091 pp/year; 0.084 pp/year, respectively) (Fig. 2e–f).

When compared to the previous PB-FW estimate^16^, local deviations are now found to occupy larger areas; for 2005, local deviation occurrence in all four subcomponents places at around 10% (Fig. 2c–f), which generally marks increase from previously reported dry and wet streamflow and soil moisture deviation occurrences between 6–10%. These differences stem especially from the underlying climate forcings of our hydrological model ensemble (“Methods”), showing increased wet streamflow deviations when comparing against the previous PB-FW estimate (Supplementary Fig. 1) and notable variation between each other (Supplementary Figs. 1–4). Moreover, although the baseline scenario uses detrended climate forcing based on reanalysis data (“Methods”), slight residual trends downwards are visible in the baseline scenario, especially for streamflow deviations (Figs. 1–2). While these differences serve as an important reminder of the dependency of PB-FW assessments on model ensemble selection, the broad trend of increasing PB-FW transgression across its subcomponents is distinguishable and robust.

At the global scale, CRF is the primary driver of PB-FW transgression (Fig. 2a, b), though its degree of dominance varies over time as well as across dry and wet streamflow and soil moisture deviations (Fig. 2c–f). CRF is almost fully responsible for increases in wet streamflow and soil moisture deviations (Fig. 2e, f), while dry deviations reflect a more mixed influence from both CRF and DHF (Fig. 2c, d). In other words, globally, CRF is driving the occurrence of both dry and wet deviations to a relatively similar degree, while DHF mostly amplifies dry deviations and has a low global-scale impact on wet deviations. This does not preclude, however, that differences between all scenarios and the baseline scenario are statistically significant (tested by Wilcoxon signed rank test^36^, two-sided test at significance level *p* = 0.05, *n* = 30) when comparing annual values over the last 30 years of our study period (1990–2019). Temporally, the divergence between CRF and DHF scenarios began to notably widen around the 1970s for all other components except for dry streamflow deviations (Fig. 2d–f), which shows how the impact of climate change has become more pronounced in the freshwater cycle during the late twentieth and early twenty-first century.

### Regional occurrence of local deviations

In addition to global PB-FW patterns (Figs. 1–2), it is essential to identify and distinguish the regional drivers of the underlying changes, since each distinct region of the world follows its unique trajectory of streamflow and soil moisture deviations. We used the HydroBASINS level 4 catchment delineation^37^ to assess these trajectories and to disaggregate the global PB-FW transgression into regional components. HydroBASINS level 4 is a hydrologically viable and sufficiently detailed zoning, which additionally allows for distinguishing between sub-catchments of larger basins (“Methods”).

**Fig. 3:**
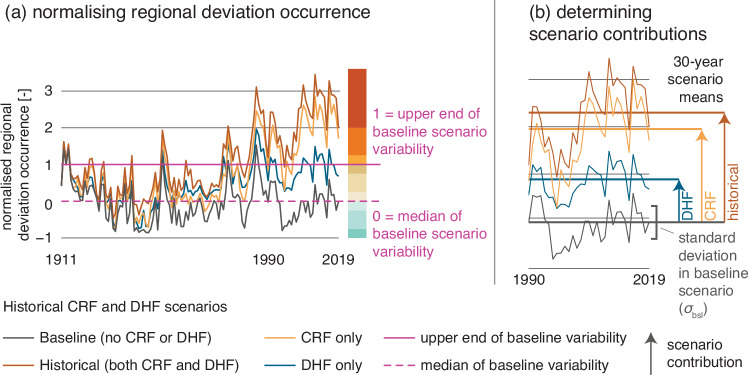
Normalising regional deviation occurrence and determining scenario contributions. Regional occurrence of local deviations is normalised with respect to the median and upper end of baseline scenario variability to compare regions with different levels of natural variation in local deviations (**a**), and different climate-related forcing (CRF) and direct human forcing (DHF) scenarios are compared to the baseline scenario to assess scenario contributions, using 30-year scenario means to account for interannual variability (**b**). Annual ensemble medians are taken for all scenarios before normalisation and scenario comparison. Steps **a**, **b** are described in detail in “Methods”.

Our regional analysis mirrors the global method: we established grid cell wise local bounds under baseline conditions, estimated the regional occurrence of local deviations for the four different scenarios and finally set region-specific (PB-like) reference boundaries at the median and upper end of baseline variability (Fig. 3a, “Methods”). While regional analysis in general was piloted already by Porkka et al.^16^, we now more thoroughly compare between regions with different levels of natural variation in local deviations by normalising the regional occurrence of local deviations relative to the two reference boundaries (Fig. 3a, “Methods”).

**Fig. 4:**
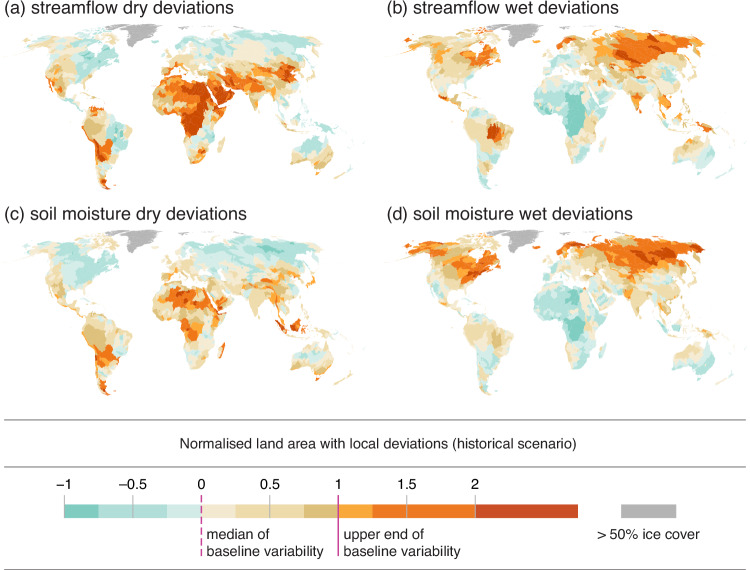
Regional occurrence of dry and wet local deviations for the historical scenario. The regional occurrence of local deviations is measured by the normalised percentage share of regional ice-free land area with local deviations, for dry streamflow deviations (**a**), wet streamflow deviations (**b**), dry soil moisture deviations (**c**), and wet soil moisture deviations (**d**). Shown is the 30-year mean (1990–2019) regional deviation occurrence, taken from annual ensemble medians to account for interannual variability (Fig. 3, “Methods”). The regions analysed here depict basins delineated by the HydroBASINS data set^37^ level 4 (*n*  = 1280, mean area 103,000 km^2^; median area 60,000 km^2^). Regional dry and wet deviation occurrence for scenarios consisting of climate-related forcing only or direct human forcing only are shown in Supplementary Figs. 5–6.

The normalised regional occurrence of local deviations varies widely across dry and wet streamflow and soil moisture deviations and 1280 regions represented in the analysis (Fig. 4). Regions with the highest occurrence of local deviations include, for example, central Africa for dry streamflow deviations (Fig. 4a), Maritime Southeast Asia for dry soil moisture deviations (Fig. 4c), and much of the northern boreal zone for wet streamflow and soil moisture deviations (Fig. 4b, d). In regions including the Congo basin and eastern Australia, for instance, the normalised occurrence of wet deviations in both streamflow and soil moisture shows negative values, suggesting less frequent instances of wet local deviations (Fig. 4b, d) and thus an overall drying pattern.

Regional research can be shown to align with these patterns; for instance, increased extreme rainfall events lead to wetting and subsequent impacts on permafrost melting and greenhouse gas emissions in the boreal zone^38^, and in the case of the Congo basin, an observed long-term drying precipitation trend and associated decline of forest productivity^39^ could be assumed to be linked with dry local deviations both in streamflow and soil moisture. Additionally, while some regions e.g. in Central Europe and western North America for dry streamflow and soil moisture deviations are yet to transgress their region-specific upper end boundaries, simply deviation occurrence does not fully capture how hydrological change is or is not occurring in the region. This is because CRF and DHF can act counterproductively, and notable human impacts on regional hydrology, such as large-scale damming in Europe^40^ or forest cover loss in North America^41^, may not turn out visible in these maps that only display the net outcome.

### Decomposition of regional CRF and DHF contributions

To assess the accumulated contributions of historical CRF and DHF on the regional occurrence of local deviations, we compared the occurrence of local deviations over the last 30 years of our study period (1990–2019) across all scenarios (Fig. 3b, “Methods”). To yield scenario contributions, we subtracted the 30-year mean of regional deviation occurrence under the baseline scenario from the 30-year mean of the three other scenarios (historical, CRF, DHF) to quantify how each scenario alone would hypothetically affect regional deviation occurrence (Fig. 3b, “Methods”). We tested for statistical significance of the scenario contribution using the Wilcoxon signed rank sum test (two-sided test, significance level *p* = 0.05, *n* = 30). Further, we categorised the scenario contributions by comparing them to regionally typical interannual variability in local deviation occurrence, quantified by standard deviation in regional deviation occurrence in the baseline scenario during years 1990–2019 (*σ*_bsl_; Fig. 3b, “Methods”). This categorisation can thus distinguish how strongly CRF and DHF have contributed to historical streamflow and soil moisture changes in each region.

**Fig. 5:**
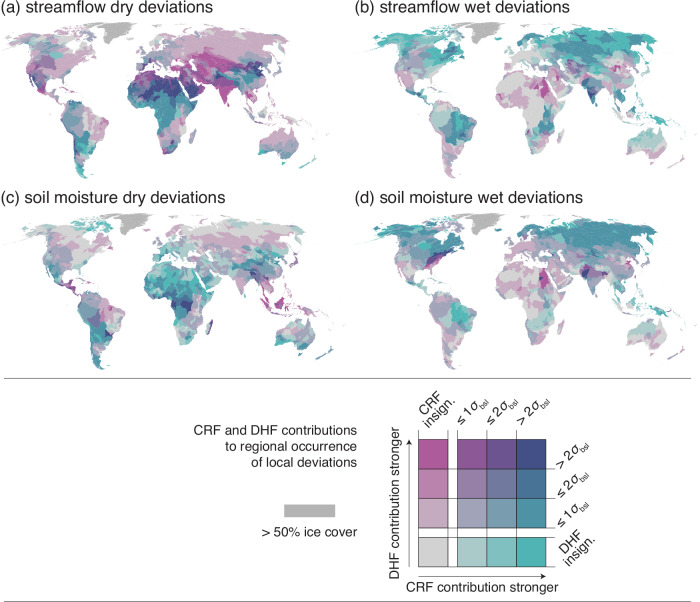
Contributions of climate-related forcing (CRF) and direct human forcing (DHF) to regional occurrence of local deviations. The CRF and DHF contributions are based on comparison between each scenario against the baseline scenario (Fig. 3b, “Methods”), for dry streamflow deviations (**a**), wet streamflow deviations (**b**), dry soil moisture deviations (**c**), and wet soil moisture deviations (**d**). Before assessing contributions, 30-year means (1990–2019) of regional deviation occurrence are taken from annual ensemble medians to account for interannual variability. Scenario contributions are tested for statistical significance using the Wilcoxon signed rank sum test (two-sided test, significance level *p* = 0.05, *n* = 30) and further mapped to a bivariate palette using regionally typical interannual variability in the baseline scenario as a reference (*σ*_bsl_; standard deviation in regional deviation occurrence under the baseline scenario during 1990–2019, insign.; insignificant) (“Methods”). The regions analysed here depict basins delineated by the HydroBASINS data set^37^ level 4 (*n*  = 1280, mean area 103,000 km^2^; median area 60,000 km^2^).

Overall, the patterns of significant CRF and DHF contributions to regional deviation occurrence (Fig. 5) are expectedly in line with globally aggregated outcomes (Fig. 2). Strong CRF contributions cover large extents and are the most widespread for wet deviations in the boreal zone (Fig. 5b, d) and for dry deviations in much of Africa (Fig. 5a, c). DHF contributes strongly to dry streamflow deviations in more confined, often agriculturally intensive regions, such as India, Central Asia and the western U.S. (Fig. 5a) and to dry soil moisture deviations in Southeast Asia (Fig. 5c). Wet deviations are primarily climate-driven, and only few locations in individual regions across North America, South and Central Asia and the Nile basin show strong DHF influence on wet deviations (Fig. 5b, d).

The spatial patterns of significant CRF and DHF contributions are, to a wide extent, polarised, because the overlap between regions experiencing the strongest influences from both CRF and DHF is relatively limited (Fig. 5). While all subcomponents show some individual regions with very strong (>2*σ*_bsl_) contributions from both CRF and DHF (Fig. 5), only dry streamflow deviations are heavily affected by contributions from both forcings across larger extents, such as northern China, the Arabian Peninsula and northern Africa (Fig. 5a). For most regions with significant CRF or DHF contributions, both forcings contribute to a net increase in the regional occurrence of deviations (Supplementary Fig. 7). However, exceptions can be found, for instance, in Central Asia and India where CRF decreases and DHF increases dry streamflow deviations (Supplementary Fig. 7a).

The regional pattern of CRF decreasing and DHF increasing dry streamflow deviations would align with climate-induced increases in dry season water availability^42^ that are, however, outweighed by substantial increases in human water demand in the same region^43^. Another contrasting pattern is in the northernmost regions of the boreal zone where CRF increases and DHF decreases wet streamflow deviations (Supplementary Fig. 7b), which may link to how under a generally wetting climate^38^, dams can still decrease wet deviations by capturing seasonal high flows^44^ that would manifest as wet deviations without the dam. Additionally, some regions are largely yet to transgress their region-specific upper end boundaries (Fig. 4), but the same regions may show comparatively strong CRF or DHF contributions (Fig. 5). This rapid and relatively recent freshwater change can be seen, for instance, in southwestern North America that has experienced recent climate-related droughts^45^ and southern China that is a region with highly growing demands for blue and green water use^46^.

### Synthesis of regional CRF and DHF contributions

**Fig. 6:**
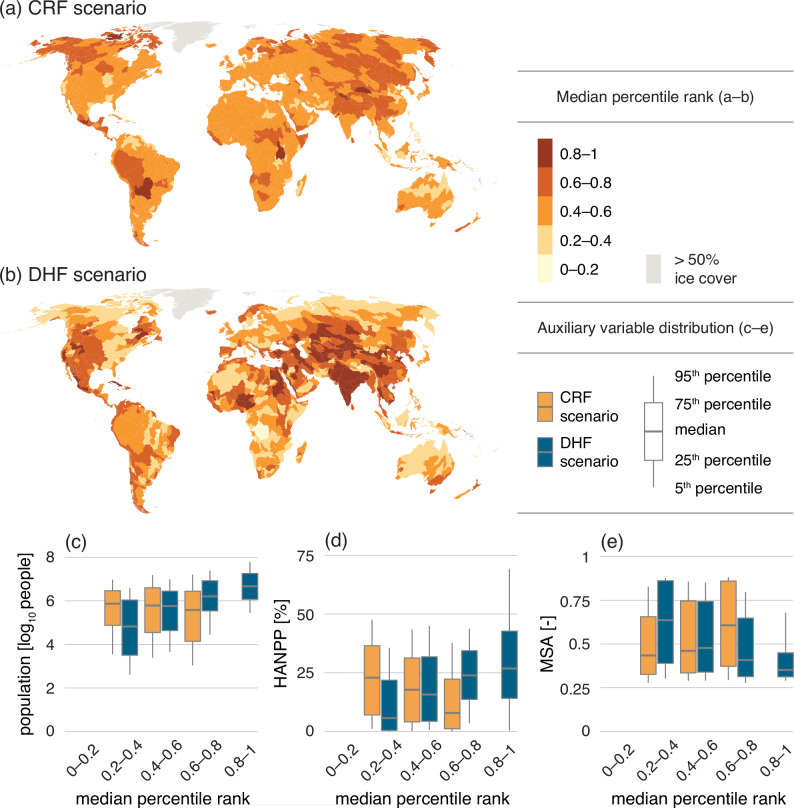
Synthesised contributions of climate-related forcing (CRF) and direct human forcing (DHF) on regional deviation occurrence together with a grouping of three auxiliary variables. For each of the four subcomponents of regional deviation occurrence (streamflow and soil moisture, dry and wet), each region is assigned a global percentile rank based on relative CRF and DHF contribution values (“Methods”). Shown here is the median of those four ranks in each region for the CRF scenario (**a**) and the DHF scenario (**b**). Median percentile rank bins in of **a**, **b** are further used to group three auxiliary variables (“Methods”): population count^47^ (**c**), human appropriation of net primary production^48^ (HANPP) (**d**), and mean species abundance^49^ (MSA) (**e**). The regions shown in **a**, **b** depict basins delineated by the HydroBASINS data set^37^ level 4 (*n*  = 1280, mean area 103,000 km^2^; median area 60,000 km^2^). Regionally aggregated population count, HANPP, and MSA are shown in Supplementary Fig. 8, and group sizes for box plots in **c**–**e** are shown in Supplementary Table 1. Box plots with group sizes less than 5% of the total number of analysed regions and box plot values outside of the 5th–95th percentile range are not shown.

To demonstrate how our approach can be used to identify particularly affected or vulnerable regions, we synthesised how CRF and DHF affect overall regional deviation occurrence by comparing their contributions in each of the four subcomponents consisting of dry and wet deviations for both streamflow and soil moisture. For each subcomponent, we ranked all regions using global percentile ranks of scenario contributions relative to interannual variability in the baseline scenario (“Methods”). Higher percentile ranks for one subcomponent in a region indicate greater contribution of CRF or DHF to increasing deviation occurrence in that subcomponent, compared to other regions. When taking the median of four independent percentile ranks across the four subcomponents—each representing a different facet of the freshwater cycle—higher median percentile ranks signal greater deviation across many subcomponents and thus greater overall contribution of CRF or DHF on pervasive systemic freshwater changes (Fig. 6). In other words, this ranking shows where climatic or direct human drivers are the most prominent across blue and green water.

In the CRF scenario, the median percentile ranks show low variance across all regions globally (standard deviation = 0.11), with higher and lower median ranks distributed across all continents (Fig. 6a). This suggests widespread but comparatively uniform historical contributions of CRF on freshwater change. In contrast, the DHF scenario has larger variance in median percentile ranks across all regions globally (standard deviation = 0.20) and shows more localised patterns. For instance, high median ranks locate to India and Central Asia, while lower median ranks are seen in the most northern latitudes and in many regions in Africa (Fig. 6b).

Finally, to open pathways for future studies by exploring how the synthesised CRF and DHF contributions align spatially with variables indicating human pressures on ecosystems, we used zonal statistics to summarise three auxiliary variables in median percentile rank bins shown in Fig. 6a, b (“Methods”). We summarised population count^47^ (Fig. 6c) and regional averages of human appropriation of net primary production^48^ (HANPP; Fig. 6d) and mean species abundance^49^ (MSA; Fig. 6e). Regions with the highest overall DHF contributions (increasing median percentile rank) seem associated with high population count but similar association between overall CRF contributions and population count appears not visible (Fig. 6c). HANPP and MSA—that both represent ecological impacts relative to undisturbed states—also show potential associations with the CRF and DHF contributions. Lower MSA, which depicts a high degree of ecosystem disturbance^49^, appears to be associated with higher DHF contributions (Fig. 6e). High HANPP seems positively associated with increasing DHF contributions and negatively associated with increasing CRF contributions (Fig. 6d). As high HANPP corresponds to a high level of human-driven alteration (e.g. by land cover change) in ecosystems’ carbon and energy flows^48^, these associations may signal how the strongest human pressures on the land surface affect both land and freshwater systems.

This synthesis suggests that the strongest overall CRF contributions may be located in regions where ecosystems might be comparatively less altered (Supplementary Fig. 8b, c), but where climate change can potentially still have a strong impact on streamflow and soil moisture (Fig. 6a). In contrast, the strongest overall DHF contributions (Fig. 6b) seem to co-occur spatially with human pressures on ecosystems (Supplementary Fig. 8b, c). Given also that strong DHF likely indicates notable human dependencies—and impacts—on freshwater resources, the regions with the strongest DHF contributions may be particularly vulnerable to further freshwater change and its adverse impacts.

## Discussion

Here, we demonstrate how the recently updated PB-FW can be used for an in-depth analysis of freshwater change and its historical drivers across global and regional scales. Our findings highlight the continued increase in PB-FW transgression over the early twenty-first century (Fig. 1), which indicates elevated freshwater-related risks in the Earth system. Although the estimates’ spread—including the previous^16^ and the current, updated hydrological model ensemble—is large, our results align with research on recent trends in specific water cycle elements showing how the water cycle is undergoing widespread and regionally variable change^3,28–30^. Projections of the water cycle^50,51^ and our specific projections of the PB-FW suggest that in the future, returning back to pre-transgression conditions appears unlikely even with ambitious climate action, but the degree and rate of further PB-FW transgression depend on the future climate change trajectory (Supplementary Text; Supplementary Fig. 9).

Our results on the historical drivers of freshwater change (Figs. 5, 6) largely agree with earlier conclusions of climatic factors affecting freshwater change across broad geographical scales^24,27^, while direct human drivers can intensify this impact in specific regions where human dependencies on freshwater are also strong^7^, especially in terms of increasingly dry conditions^23,25^. Our consequent exploratory analysis, which appears to suggest alignment between freshwater change and human pressures on ecosystems (Fig. 6), may additionally serve as an early-stage validation for our method in distinguishing particularly vulnerable regions. Therefore, our analysis can point to prospects for further studying freshwater change and vulnerabilities to its impacts in specific regions, along with a globally comparable characterisation of the freshwater change that each region is going through (e.g. blue vs. green water, dry vs. wet deviations). Regional implications made with plausible confidence, however, remain to require additional, case-dependent research and validation. Nevertheless, our regional results are directly compatible with the PB-FW, showing the high degree of spatial granularity underlying its global transgression. We thus believe that our results contribute to bridging the scales between the PBs framework, which is largely focused on the broad Earth system, and regional-scale research, which may allow for more tangible points and means of action to practically address freshwater change.

Interpreting the PBs framework in light of our findings emphasises the interconnectedness of Earth system processes represented by individual PBs^52,53^. Even in the hypothetical absence of DHF, CRF alone would have caused a notable PB-FW transgression (Fig. 2). Given that a separate PB for climate change (PB-CC) exists^18^, PB-FW could be argued to be redundant because its transgression is highly conditional to PB-CC transgression. We, however, conversely argue that the PB-FW can distinguish factors that the PB-CC cannot; the PB-CC cannot separate between widely variable regional patterns of the climate change impact on blue and green water (Supplementary Fig. 5), and in some regions, non-climatic factors may be much stronger contributors to freshwater change (Fig. 5). Similarly, the first PB-FW approaches that only considered human blue water consumption^2,22^ and their further applications^26,54,55^ neglect most of these factors (and the systemic influence of climate) and thus remain limited in how they can be used to understand freshwater-related risks in the Earth system. Therefore, in accordance with previous studies^1,32^, we strongly advocate for considering the freshwater cycle as a highly dynamic Earth system component instead of a controllable tap. Similar applications and appraisals of other PBs, as well, could aid in addressing critiques of the framework^56^ and in broader goals of using the framework to inform sustainable environmental governance and Earth system stewardship^31,57,58^.

Our approach has some key limitations that should be acknowledged. Numeric uncertainties stem largely from the simulated coarse scale data, although the models producing those data are as advanced as possible for a global-scale analysis (“Methods”) and first comprehensive validations of the ISIMIP 3a data are currently emerging^59,60^. Adopting the ensemble modelling approach can mitigate these uncertainties to an extent (“Methods”), but continued development and updates of the PB-FW analysis following the emergence of new models, data and software^61^ remains a recurring task. Moreover, separating increasingly precise human drivers, such as locally specific land and water management strategies (e.g. irrigation), was limited by the current ISIMIP data.

Perhaps most importantly, however, linking indicators that depict frequencies of anomalous streamflow and soil moisture conditions with an elevated level of freshwater-related Earth system risks is a general presumption stemming from the PBs framework^16–18^. Due to the absence of systematic studies to reflect local and regional variation in the levels of freshwater change that translate to unacceptable Earth system risks, both grid cell-specific local bounds (5th–95th percentile bounds in streamflow and soil moisture) and regionally aggregated thresholds (95th percentile of local deviation occurrence in the baseline scenario) are set in a globally uniform manner (“Methods”). In general, it is notably difficult to devise globally applicable indicators with clear implications when transgressed, while also comprehensively representing highly complex Earth system processes, such as the freshwater cycle. Gathering systematic, empirically grounded evidence to evaluate these presumptions, and possibly adjusting local, regional and global boundaries based on that evidence remains a priority for improving the conceptualisation and quantification of cascading impacts and Earth system risks due to freshwater change.

Improved understanding of global freshwater change and the interplay between CRF and DHF that drive its regional features enhances the capacity to assess, mitigate, or possibly adapt to its adverse impacts on the Earth system and societies. Although our approach is a step towards developing these capacities, many more steps towards a holistic approach remain to be taken. The here-shown biophysical change and its drivers should additionally be connected with the higher-degree and system-specific telecoupled drivers of change^62^. For instance, regions with the highest historical changes in streamflow and soil moisture—that are often amplified by DHF—may disproportionately contribute and be exposed to freshwater-related Earth system risks. At the same time, high DHF contributions signal sustained dependence and impact on blue and green water. The external, non-biophysical drivers of that dependence (e.g. trade markets^63^) may, however, be located far from the biophysical change location, which complicates how human decisions ultimately affecting local and regional water resources are made^64,65^. Therefore, coupling these initial driving forces to the pathways ending in freshwater-related Earth system risks would be one of the most important next steps. Advancing this line of research could weave together the PB framework’s biophysically oriented global scale thinking with more holistic and societally actionable deliberation of the drivers and impacts of freshwater change in the Earth system.

## Methods

### Climate-related forcing and direct human forcing scenarios

Our main data source was the ISIMIP data repository^34^, from which we used data from the ISIMIP simulation round 3a experiments^33^. Hydrological data in the ISIMIP 3a experiments are simulated by GHMs using reanalysis-based CRF and dynamic DHF. CRF describes atmospheric variables, such as temperature and precipitation, which affect water availability and partitioning on the land surface. Some of the most important DHF variables include, for instance, land cover and use, cropping patterns, water abstraction, and flow regulation by dams and reservoirs, which further affect how water is distributed on the land surface and belowground.

To attribute the impact of CRF and DHF on hydrological change, we required four distinct simulation scenarios: (1) the baseline scenario with CRF derived from detrended historical climate reanalysis data and DHF at 1901 levels, (2) the historical scenario consisting of both historical CRF and DHF, (3) the CRF scenario with DHF at 1901 levels, and (4) the DHF scenario with CRF derived from detrended historical climate reanalysis data^33^. The CRF detrending procedure, done by ISIMIP for the simulation rounds’ input data, removes the global climate change trend from historical climate reanalysis data, but retains their internal variability, thus allowing the detrended climate data set to be used for climate change impact attribution^33,66^. While year 1901 levels do not mean a complete absence of DHF, the baseline scenario represents conditions that are largely absent from major anthropogenic modifications of the water cycle^33^. These conditions are thus adequately representative of pre-industrial-like reference conditions desired for the planetary boundaries framework^18^, although they slightly differ from the nominally pre-industrial reference conditions used by Porkka et al.^16^ for their PB-FW analysis.

GHMs participating in ISIMIP simulations are land surface models, which restricts the representation of hydrological impacts to the land surface (e.g. runoff propagation) and belowground (e.g. subsurface flows), leaving out land-atmosphere interactions (e.g. rainfall regulation by evapotranspiration) that would require coupling the GHMs with atmospheric models^67^. The GHMs participating in ISIMIP experiments, however, often have long legacies of extensive model development and validation^68–70^, and the first validations of ISIMIP 3a hydrological data are continuously emerging^59,60^, which is why we chose to leave an explicit validation outside of the scope of this study. Instead, we use the common and often adequate^71,72^ ensemble modelling approach to mitigate uncertainties stemming from GHMs being subject to various process simplifications and variance in how hydrological processes within them are implemented^73^.

### Model ensemble selection

In accordance with Porkka et al.^16^, we used streamflow as the control variable for blue water change and root-zone soil moisture (hereinafter soil moisture) as the control variable for green water change. Initially, we selected ISIMIP 3a simulation outputs from all GHMs for which outputs were available in all four required scenarios (baseline, historical, CRF, DHF). This yielded six GHMs available for streamflow (H08^74^, HydroPy^75^, JULES-W2^76^, LPJmL5-7-10-fire^77^, MIROC-INTEG-LAND^78^ and WaterGAP2^69^) and four GHMs available for soil moisture (HydroPy, LPJmL5-7-10-fire, MIROC-INTEG-LAND and WEB-DHM-SG^79^). Although available in the ISIMIP repository, we did not include JULES-W2-DDM30 for streamflow because the only difference to JULES-W2 is in the flow routing model implementation.

Simulations forced with three CRF data sets (GSWP3-W5E5, 20CRv3-ERA5 and 20CRv3-W5E5)^33,80^ were available for models H08, WaterGAP2-2e, LPJmL5-7-10-fire and MIROC-INTEG-LAND, whereas simulations using one CRF data set (GSWP3-W5E5) were available for HydroPy, JULES-W2 and WEB-DHM-SG. Additionally, although simulations using another CRF data set (20CRv3) were available, we did not select them because they ended in 2015, while data simulated with the other CRF data sets ended in 2019 or 2021^33^. Simulations with CRF from 20CRv3-ERA5 would have provided data until 2021, but to be consistent with 20CRv3-W5E5 and GSWP3-W5E5, we discarded the two last years from them.

Our analysis period thus consistently covered years 1901–2019 for all selected ensemble members (Supplementary Table 2). The spatial resolution of the gridded hydrological data was 0.5 degrees (corresponding up to approximately 55 × 55 km when at the Equator). Both streamflow and soil moisture data were delivered at a monthly time resolution, with streamflow in units m^3^/s, and soil moisture in units kg/m^2^. Streamflow data and most soil moisture data required no further pre-processing, but soil moisture data simulated by LPJmL5-7-10-fire were provided in five depth layers, which were summed to yield total soil moisture. For 1–3 ensemble members, the data, when gathered from ISIMIP, contained sporadic instances of missing or negative monthly values, which were filled with the mean of all non-missing and non-negative values of the respective grid cell, month and simulation scenario. Since up to 380 grid cells (out of 67,420) needed infilling for soil moisture, and the only case of missing data was found for an individual month of streamflow data, we assume that the infilling has a negligible impact on our results.

When performing our primary global analysis (Fig. 1) for each ensemble member separately, we found that for some ensemble members, the baseline and historical scenarios were mismatched in the beginning of the twentieth century (Supplementary Figs. 10, 11). This was the case for streamflow simulations from H08 and MIROC-INTEG-LAND forced with GSWP3-W5E5 (Supplementary Fig. 10c, k) and for soil moisture simulations from all LPJmL5-7-10-fire ensemble members (Supplementary Fig. 11b–d) and from MIROC-INTEG-LAND forced with GSWP3-W5E5 (Supplementary Fig. 11g). We assume that this behaviour stems from differences in scenario simulations’ input data, because with CRF and DHF at early twentieth century levels, all scenarios would be expected to be in near agreement with the baseline scenario. Given that we had no access to these input data, we decided to discard these ensemble members from the presented main results. However, global results (equivalent to Fig. 2) including also the discarded ensemble members are shown in Supplementary Fig. 12.

After determining the maximally large GHM ensemble and filtering it by the above listed constraints and consistency checks, the final resulting ensemble sizes were *n* = 12 for streamflow and *n* = 4 for soil moisture. The final streamflow data ensemble consisted of simulations from six different GHMs forced with three CRF data sets with equal counts (4 ensemble members per CRF data set), whereas the final soil moisture data ensemble consisted of simulations from three different GHMs forced with three CRF data sets with 1, 1, and 2 ensemble members each (Supplementary Table 2). Thus, the selected model ensembles were not substantially biased towards individual GHMs or CRF data sets and they can be thought to capture a decent amount of model and forcing variance to provide robust results.

The selected GHM ensembles differ from those used by Porkka et al.^16^. Most notably, the CRF data sets used for ISIMIP 3a simulations are based on historical climate reanalysis instead of modelled climate from general circulation models. This appears to affect especially the degree of large-scale wetting, which is visible when comparing estimates based on two different versions of streamflow data, both run with WaterGAP2 (Supplementary Fig. 1). Some updates in DHF (e.g. population data) have also been made for ISIMIP 3a compared to the previously used ISIMIP 2b, but, for instance, land use patterns are identical between the two experiment rounds^33^. While not all GHMs are present in both studies, even those that are (H08, LPJmL, WaterGAP) use newer versions in ISIMIP 3a experiments; for instance, WaterGAP2-2e used in ISIMIP 3a has an updated database of lakes and reservoirs compared to previous versions^69^.

### Occurrence of local deviations

In determining the occurrence of local deviations—i.e. representing aggregate human impacts on the freshwater cycle globally and regionally—we closely followed the methodological framework conceptualised by Wang-Erlandsson et al.^17^ and scrutinised by Porkka et al.^16^. With main methodological choices corresponding to Porkka et al.^16^, global results (i.e. PB-FW transgression estimates) between the two are directly comparable, although subject to differences in the used hydrological data as outlined above.

We first established local variability bounds for each grid cell, month and ensemble member, separately for streamflow and soil moisture. The grid cell specific 5th and 95th percentile values in the baseline scenario were defined as the local bounds. Here, we assume that increasing frequencies of anomalous streamflow and soil moisture conditions outside of the local bounds expose local environments to rare or unforeseen conditions that they might not be adapted to, which may further relate to adverse impacts in that local environment^16^. To avoid potential traces of GHM simulation spinup (i.e. time cycles required to run a model before hydrological stores reach an equilibrium) affecting the local bounds (exemplified e.g. in Supplementary Fig. 10a, b), we discarded years 1901–1910 from determining the local bounds and from all subsequent steps. Second, we detected grid cell scale local deviations, i.e. monthly events of streamflow or soil moisture values falling outside of the local bounds. Monthly values below the local 5th percentile bound were marked as dry local deviations and monthly values above the local 95th percentile bound were marked as wet local deviations. The grid cell scale local deviations were determined in a binary fashion, which means that we did not further specify the local deviations by how far from the respective local bound each dry or wet local deviation fell, but treated the local deviations simplistically as monthly events of deviation or no deviation.

We estimated the global and regional occurrence of local deviations by aggregating them within land areas. For each month and ensemble member, and separately for streamflow and soil moisture, we summed up physical land areas of grid cells that were marked as local deviations, yielding land area with dry local deviations (Fig. 2c, d) and land area with wet local deviations (Fig. 2e, f); these two land areas were further summed to yield land area with dry and wet local deviations (Fig. 1, Fig. 2a, b). The three sums of land area with local deviations were related to the total global or regional land area, yielding percentage shares. Given that the local variability bounds were limited by the 5th–95th percentile range, expectedly 5% of all values were marked as dry local deviations and 5% as wet local deviations for each grid cell, month and ensemble member in the baseline scenario. Thus, also the expected global percentage of land area with local deviations was approximately 5% for dry and wet deviations in the baseline scenario, respectively.

Grid cells covered by permanent land ice were excluded from the land area aggregations using the History Database of the Global Environment (HYDE) version 3.5 anthrome classification^47^. The regional aggregation of local deviations additionally considered region boundaries that cut across coarse 0.5-degree grid cells, considering only the grid cell fraction that lies within the region boundary^81^; thus, the regional land area with local deviations could never exceed the total regional land area.

Finally, we used the occurrence of local deviations in the baseline scenario to demarcate two reference boundaries: the median of baseline variability and the upper end of baseline variability. Both reference boundaries were set at the global scale and for each region separately, allowing the regional reference boundaries to scale based on the variance of local deviation occurrence in the baseline scenario. Before setting the reference boundaries, we took annual means of monthly percentages of land area with local deviations for dry deviations, wet deviations and for dry and wet deviations; all for each ensemble member separately. Taking annual means emphasises temporally extensive changes in streamflow and soil moisture since annual mean values are higher for years during which local deviations occur over multiple months.

Annual means were followed by taking ensemble medians of the three percentage shares, resulting in three time series (*n* = 109, over years 1911–2019) that described annual percentages of land area with dry, wet and dry and wet local deviations. For each of these time series, the median value was defined as the median of baseline variability, and the 95th percentile value was defined as the upper end of baseline variability. Here, following Porkka et al.^16^ and the PBs framework^18^, we presume that widespread (regional or global) simultaneous occurrence of anomalous conditions beyond pre-industrial-like baseline conditions contributes to or may trigger abrupt or gradual systemic shifts, which, consequently, contribute to freshwater-related risks in the Earth system. We chose the upper end of baseline scenario variability as a precautionary reference boundary to represent these risks, since it demarcates conditions that occurred rarely (expectedly once in every 20 years) under water cycle conditions that were yet largely undisturbed by direct and indirect human pressures. At the global scale, the upper end of baseline variability for dry and wet local deviations corresponds to the PB-FW^16,18^.

To compare the three other scenarios (historical, CRF, DHF) against the baseline scenario, we repeated the detection of local deviations against local variability bounds set by the baseline scenario, followed by estimation of local deviation occurrence by aggregating local deviations both globally and regionally. Annual means were taken similarly from monthly percentage shares of land area with deviations, followed by taking ensemble medians. Local variability bounds and the median and the upper end of baseline variability were set only once globally and for each region, and all scenarios were thus compared against the same baseline. Growth rates for global deviation occurrence were estimated by linear Theil-Sen regression slopes, significance of which was tested with the Kendall test (two-sided test, significance level *p* = 0.05), using the R package zyp^35^.

### Regional normalisation of deviation occurrence

We estimated the regional occurrence of local deviations within HydroBASINS level 4 catchment boundaries^37^, which are hierarchically nested sub-catchments delineated based on flow directions. The hydrological viability and the consistently detailed intermediate resolution made it a good choice for our analysis. Out of total 1341 catchments in the original HydroBASINS level 4 polygon layer, 41 had more than 50% permanent ice cover and 20 did not overlap with hydrological data from ISIMIP. Thus, the final count of analysed regions was 1280, with mean area of 103,000 km^2^ and median area 60,000 km^2^. While regions with more than 50% permanent ice cover were omitted from the regional analysis, non-ice grid cells within those regions were considered when aggregating the global land area with local deviations.

To compare the occurrence of local deviations across differently sized regions with variable ambient CRF and DHF, we normalised the regional land area with local deviations with respect to the median and upper end of baseline variability (Fig. 3a), according to Eqs. 1–3. The normalised local deviation occurrence yields values in the interval [–1, $\infty$], and particularly value zero when local deviation occurrence corresponds to the region-specific median of baseline variability and value one when local deviation occurrence is at the region-specific upper end of baseline variability.

$$\begin{array}{cc}{{{\rm{condition}}\,{{\rm{for}}\, }}}{{{{\rm{areaLocDev}}}}}_{s,{y}} & {\rm{resulting}}\; {{{{\rm{areaLocDev}}}}}_{s,{y},{{{\rm{norm}}}}}\end{array}$$

1 $$\begin{array}{cc}{{{\rm{if}}}}{{{{\rm{areaLocDev}}}}}_{s,{y}} < {{{{\rm{Med}}}}}_{{{{\rm{bsl}}}}} &=\frac{{{{{\rm{areaLocDev}}}}}_{s,{y}}\,-\,{{{{\rm{Med}}}}}_{{{{\rm{bsl}}}}}}{{{{{\rm{Med}}}}}_{{{{\rm{bsl}}}}}}\end{array}$$

2 $$\begin{array}{cc}{{{{\rm{if\; Med}}}}}_{{{{\rm{bsl}}}}}\le {{{{\rm{areaLocDev}}}}}_{s,{y}}\le {{{{\rm{UpEnd}}}}}_{{{{\rm{bsl}}}}} &=\frac{{{{{\rm{areaLocDev}}}}}_{s,{y}}-\,{{{{\rm{Med}}}}}_{{{{\rm{bsl}}}}}}{{{{{\rm{UpEnd}}}}}_{{{{\rm{bsl}}}}}\,-\,{{{{\rm{Med}}}}}_{{{{\rm{bsl}}}}}}\end{array}$$

3 $$\begin{array}{cc}{{{\rm{if}}}}{{{{\rm{areaLocDev}}}}}_{s,{y}} > {{{{\rm{UpEnd}}}}}_{{{{\rm{bsl}}}}} &=1+\frac{{{{{{\rm{areaLocDev}}}}}_{s,{y}}-{{{\rm{UpEnd}}}}}_{{{{\rm{bsl}}}}}}{{{{{\rm{UpEnd}}}}}_{{{{\rm{bsl}}}}}}\end{array}$$

In Eqs. 1–3, ${{{{\rm{areaLocDev}}}}}_{s,{y}}$ refers to a regional percentage of land area with local deviations in scenario $s$ for year $y$, ${{{{\rm{Med}}}}}_{{{{\rm{bsl}}}}}$ to the region-specific median of baseline variability, ${{{{\rm{UpEnd}}}}}_{{{{\rm{bsl}}}}}$ to the region-specific upper end of baseline variability and ${{{{\rm{areaLocDev}}}}}_{s,{y},{{{\rm{norm}}}}}$ to the normalised regional land area with local deviations in scenario $s$ for year $y$.

Normalised regional deviation occurrence shows how close to the reference boundaries deviation occurrence in each scenario is in each region. Additionally, increasing local deviation occurrence in regions with high natural variance in the baseline scenario does not affect the normalised metric as much as the same amount of increasing absolute (percentage point) local deviation occurrence in regions with lower natural variance in the baseline scenario. To further account for interannual variability, we took 30-year means of the normalised regional land areas with local deviations over the last 30 years of our study period (1990–2019) before mapping them (Fig. 4, Supplementary Figs. 3–6).

### Contributions of CRF and DHF scenarios

We further compared the historical, CRF, and DHF scenarios against the baseline scenario to determine how much each scenario contributes to regional deviation occurrence (Fig. 3b). We first took the 30-year mean (to account for interannual variability) of regional deviation occurrence in the baseline scenario over 1990–2019 (Eq. 4), followed by determining scenario contributions, i.e. differences between each scenario and the baseline scenario, using 30-year means of regional deviation occurrence also for the other scenarios (Eq. 5). We tested for statistical significance of the scenario contribution using the Wilcoxon signed rank sum test (two-sided test, significance level *p* = 0.05) from R package stats^36^, applied for annual values over 1990–2019 (*n* = 30). Additionally, we used the standard deviation of regional deviation occurrence in the baseline scenario (Eq. 6), taken similarly over 1990–2019 as the mean, as a benchmark value to further categorise scenario contribution strengths in Fig. 5.

4 $${\mu }_{{{{\rm{bsl}}}}}=\frac{1}{30}\mathop{\sum}\limits_{{y}=\,1}^{30}{{{{\rm{areaLocDev}}}}}_{{{{\rm{bsl}}}},\,1990+(y-1)}$$

5 $${{{{\rm{cntr}}}}}_{s}=(\frac{1}{30}\mathop{\sum}\limits_{y\,=\,1}^{30}{{{{\rm{areaLocDev}}}}}_{s,\,1990+(y-1)})-\,{\mu }_{{{{\rm{bsl}}}}}$$

6 $${\sigma }_{{{{\rm{bsl}}}}}=\sqrt{\frac{1}{30}\mathop{\sum}\limits_{y\,=\,1}^{30}{({{{{\rm{areaLocDev}}}}}_{{{{\rm{bsl}}}},\,1990+(y-1)}-{\mu }_{{{{\rm{bsl}}}}})}^{2}}$$

In Eqs. 4–6, ${{{{\rm{areaLocDev}}}}}_{s,{y}}$ refers to a regional percentage of land area with local deviations in scenario $s$ for year $y$, and ${{{{\rm{cntr}}}}}_{s}$ represents a contribution from scenario $s$. Since normalised deviation occurrence can be non-linear, being scaled according to the two reference boundaries (Eqs. 1–3), we used non-normalised percentages of land area with local deviations (${{{{\rm{areaLocDev}}}}}_{s,{y}}$) to assess scenario contributions.

The scenario contributions allowed us to compare how strongly CRF and DHF increase regional occurrence of local deviations. In comparing contribution strengths (Fig. 5), we only considered increasing regional occurrence of deviations; contributions towards decreasing regional occurrence of deviations were not analysed. However, scenario contributions (Eq. 5) could be negative, which allowed us to evaluate whether both CRF and DHF increase regional deviation occurrence or if one of them significantly increases and one of them significantly decreases regional deviation occurrence (Supplementary Fig. 7).

### Synthesis of CRF and DHF contributions

We synthesised our analysis of CRF and DHF contributions on regional deviation occurrence across four distinct subcomponents—dry and wet deviations for both streamflow and soil moisture (Fig. 6). The synthesis consisted of first assigning global percentile ranks for CRF and DHF contributions in each of the four subcomponents and then taking the median of these four ranks, separately for each region and the CRF and DHF scenarios. Prior to percentile ranking, CRF and DHF scenario contributions were related to the standard deviation of regional deviation occurrence in the baseline scenario (Eq. 7) to account for region-specific natural variability.

7 $${{{{\rm{cntr}}}}}_{s,{{{\rm{subComp}}}},{{{\rm{relative}}}}}=\frac{{{{{\rm{cntr}}}}}_{s,{{{\rm{subComp}}}}}}{{\sigma }_{{{{\rm{bsl}}}}}}$$

In Eq. 7, ${\sigma }_{{{{\rm{bsl}}}}}$ is yielded from Eq. 6 and ${{{{\rm{cntr}}}}}_{s,{{{\rm{subComp}}}}}$ from Eq. 5 for CRF and DHF scenarios and the four subcomponents of dry and wet deviations for both streamflow and soil moisture. Percentile ranks were subsequently assigned for each ${{{{\rm{cntr}}}}}_{s,{{{\rm{subComp}}}},{{{\rm{relative}}}}}$ across all regions (*n* = 1280), and, for each region, the median of the four percentile ranks for four subcomponents was taken to represent the overall contribution of CRF or DHF on systemic freshwater change.

Furthermore, we binned the median CRF and DHF contribution percentile ranks to five bins and summarised three auxiliary variables within them (Fig. 6c–e). The variables consisted of year 2019 population count from the HYDE 3.5 database^47^, year 2010 human appropriation of net primary production^48^ (HANPP), and year 2015 mean species abundance^49^ (MSA). All variables were aggregated regionally before summarising them. Prior to regional aggregation, population count and MSA required no pre-processing. HANPP as a percentage of net primary production (NPP) was formed by summing the three HANPP components (harvest, land use change, and deforestation) and relating the sum to potential NPP to yield percentage shares, in accordance with the HANPP framework^48^. In regional aggregation, population count was summed from grid cells overlapping with each region, whereas HANPP (percentage) and MSA (unitless) were aggregated regionally using grid cell area weighted averages. All operations considered only the grid cell fractions that were within the region boundaries^81^ and weighted the population sum or regional average accordingly.

### Reporting summary

Further information on research design is available in the Nature Portfolio Reporting Summary linked to this article.

## Supplementary information

Supplementary information

Reporting Summary

Transparent Peer Review file

## Data Availability

Output data produced by our analysis are deposited in a public database available at 10.5281/zenodo.19663530.

## Code availability

The code used in producing the results shown in this study is publicly available at https://github.com/vvirkki/pb-fw-drivers.
