# Supplementary material for: Regionally divergent drivers behind transgressions of the freshwater change planetary boundary
Source: Nat Commun. 2026 May 13;17:5132. doi: 10.1038/s41467-026-73051-x (PMC13250156; doi:10.1038/s41467-026-73051-x)
Supplement: Supplementary file 1 — Supplementary information [file 41467_2026_73051_MOESM1_ESM.pdf]

Supplementary information for

Regionally divergent drivers behind transgressions of the freshwater change planetary boundary

This supplementary information includes:

Supplementary Text
Supplementary Figures 1–12
Supplementary Tables 1–2
Supplementary References

Supplementary Text

Projecting global freshwater deviation occurrence to the future. Given the increasing contribution of climate-related forcing (CRF) in determining the status of the planetary boundary for freshwater change (PB-FW) (Fig. 2), we assessed projected future trajectories of global streamflow and soil moisture deviation occurrence under three climate change scenarios. The Inter-Sectoral Impact Model Intercomparison Project (ISIMIP) simulation round 3a data[1] covers historical simulations ending in the year 2019 (Methods), which is why for future projections, we switched to data from ISIMIP simulation round 3b experiments spanning years 1850–2100 in total[2,3]. Differing from ISIMIP 3a, which uses CRF based on historical reanalysis, data simulated by ISIMIP 3b experiments use CRF from climate reconstructions and projections derived from general circulation models (GCMs)[3].

We re-established the counterfactual baseline scenario using ISIMIP 3b data to avoid comparing hydrological simulations that would have different CRFs from reanalysis and GCMs. In the ISIMIP 3b baseline scenario, CRF is fixed at levels representing pre-industrial climate conditions (CO_2 and CH_4 concentrations at year 1850 levels), and direct human forcings (DHF) are equally fixed at year 1850 levels[3]. In historical simulations for years 1850–2014, both CRF and DHF follow historical changes[3]. For projecting future climate change impacts on global freshwater deviation occurrence for years 2015–2100, we used three scenarios comprising SSP1–RCP2.6 with low CO_2 emissions, SSP3–RCP7.0 with medium-to-high CO_2 emissions, and SSP5–RCP8.5 with high CO_2 emissions[3]. All future scenarios used fixed DHF at year 2015 levels, which means that possible DHF adaptation measures were not considered in the analysis, and the results display only the projected impact of future climate change.

We selected ISIMIP 3b simulation outputs from all global hydrological models (GHMs) for which outputs were available in all five required scenarios (baseline, historical, SSP1–RCP2.6, SSP3–RCP7.0, SSP5–RCP8.5). This yielded three GHMs available for streamflow (H08[4], MIROC-INTEG-LAND[5], and WaterGAP2[6]) and one GHM available for soil moisture (MIROC-INTEG-LAND). For all models, simulations forced with five GCM-based CRF data sets were available (from GFDL-ESM4, IPSL-CM6A-LR, MPI-ESM1-2-HR, MRI-ESM-2-0, and UKESM1-0-LL)[3,7]. The resulting ensemble sizes for future projections were thus n = 15 for streamflow and n = 5 for soil moisture.

Besides using ISIMIP 3b data, we otherwise performed the projections of global freshwater deviation occurrence similarly as for ISIMIP 3a data (Methods). We established local variability bounds (between 5th–95th percentile values) for each grid cell, month, and ensemble member, separately for streamflow and soil moisture, using years 1860–2014 from the baseline scenario. We then detected local deviations and aggregated them globally to yield monthly percentage shares of ice-free land area with local deviations. Annual means were taken from the monthly percentage shares, followed by taking ensemble medians. Ensemble median annual time series of land area with local deviations under the baseline scenario were finally used to set the median and upper end of baseline variability, using years 1860–

2014, and thus making the reference boundaries specific to the ISIMIP 3b counterfactual baseline scenario. This allowed for side-by-side comparison between historical and future projected global deviation occurrence. By the re-establishment of the baseline scenario and different hydrological model ensemble, however, the historical values were not directly comparable to those derived from ISIMIP 3a data.

Persistently continued deviations in the freshwater cycle. Projecting the global streamflow and soil moisture deviation occurrence to year 2100 shows mostly an increasing PB-FW transgression trajectory (Supplementary Fig. 9). While the higher-emission scenarios SSP3–RCP7.0 and SSP5–RCP8.5 point towards a persistent increase in global deviation occurrence across all subcomponents of the PB-FW, the low-emission SSP1–RCP2.6 scenario is projected to stabilise (Supplementary Fig. 9a, c–e) or begin declining (Supplementary Fig. 9b, f) the occurrence of local deviations. In 2100, streamflow deviations are projected to cover 26.2% (~34 million km^2) of ice-free land area in the SSP1–RCP2.6 scenario and 42.6% (~55.4 million km^2) in the SSP5–RCP8.5 scenario, whereas these figures are 28.3% (~36.8 million km^2) in the SSP1–RCP2.6 scenario and 40.2% (~52.3 million km^2) in the SSP5–RCP8.5 scenario for soil moisture, respectively. Despite the large scenario spread, the distinct shapes of the trajectories demonstrate the stabilising effect of the SSP1–RCP2.6 low-emission pathway, whereas higher-emission scenarios point towards continuously increasing PB-FW transgressions (Supplementary Fig. 9).

When comparing global freshwater deviation occurrence based on ISIMIP 3a simulations (reanalysis-based CRF; Fig. 2) and ISIMIP 3b simulations (GCM-based CRF; Supplementary Fig. 9), historical dry streamflow and soil moisture deviation occurrences are in relatively fair agreement, with year 2015 values around 13–15% (Fig. 2c–d; Supplementary Fig. 9c–d). However, 20th century wet deviation occurrence trajectories, especially for streamflow, are more divergent between results yielded from ISIMIP 3a and 3b simulations (Fig. 2e–f; Supplementary Fig. 9e–f), with ISIMIP 3b using GCM-based CRF showing less wet deviation occurrence. This could link to the divergent large-scale wetting signals between reanalysis-based and GCM-based CRFs, which is also seen in comparison of global streamflow deviation occurrence between ISIMIP 3a simulations and another set of global deviation occurrence estimates based on GCM-forced ISIMIP simulation round 2b experiments[8] (Supplementary Fig. 1). It should, additionally, be noted that while the baseline scenario appears trendless when using ISIMIP 3b data, traces of model spinup and discontinuities between historical and future scenarios are clearly visible around the year 2015 breakpoint between scenarios (Supplementary Fig. 9), which likely has a distorting effect on precise numerical estimates.

While the freshwater cycle has already been persistently altered and future climate change projections show how returning to conditions preceding PB-FW transgression appears unlikely, the degree and rate of further change remain dependent on climate change pathways. Notwithstanding large uncertainties and mismatches between simulations in the ISIMIP 3b ensemble, the ambition level of climate action

can possibly stabilise the PB-FW transgression trajectory – or continuously increase it, should CO_2 emissions remain at a high level. In future studies, increasing model ensemble sizes with the ongoing yet incomplete ISIMIP 3b experiment round[3], correcting apparent mismatches at the breakpoint between historical and future scenarios, adding DHF adaptation scenarios, and extending the analysis to cover regionally explicit aspects would be warranted for a more elaborate outlook on future PB-FW projections.

Supplementary Figures

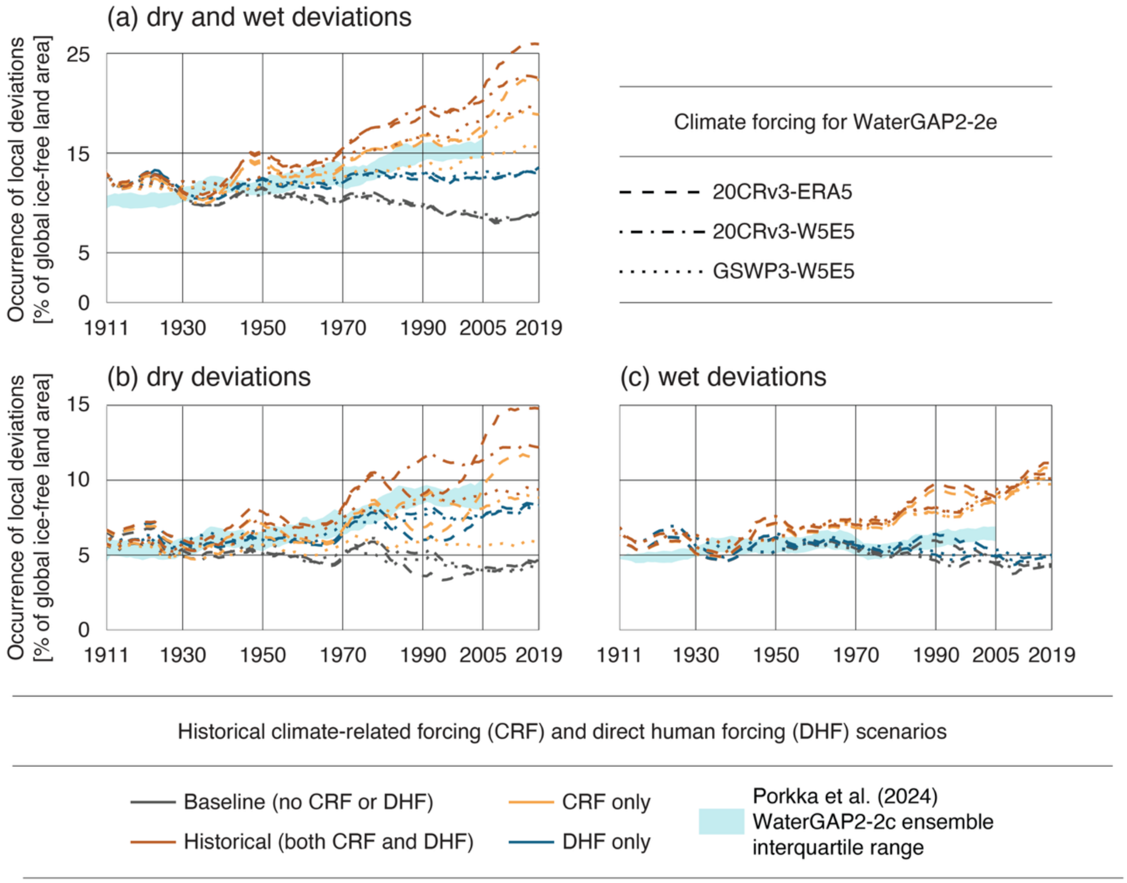

Supplementary Figure 1. Global occurrence of dry and wet local streamflow deviations under different climate-related forcing (CRF) and direct human forcing (DHF) scenarios, separately for three WaterGAP2-2e simulations using three different CRF data sets. The global occurrence of local streamflow deviations is measured by the percentage share of global ice-free land area with local deviations, for dry and wet deviations (a), dry deviations (b), and wet deviations (c). Shown is the annual percentage, which is computed as an average of monthly percentages (Methods). Time series of the occurrence of local deviations and limits of the ensemble interquartile range (IQR) are smoothed with a 10-year moving (trailing) mean over the annual percentage and ensemble IQR limits, respectively. Benchmark values until year 2005 are taken from the previous PB-FW estimate[8], specifically for WaterGAP2-2c forced with CRF from four general circulation models.

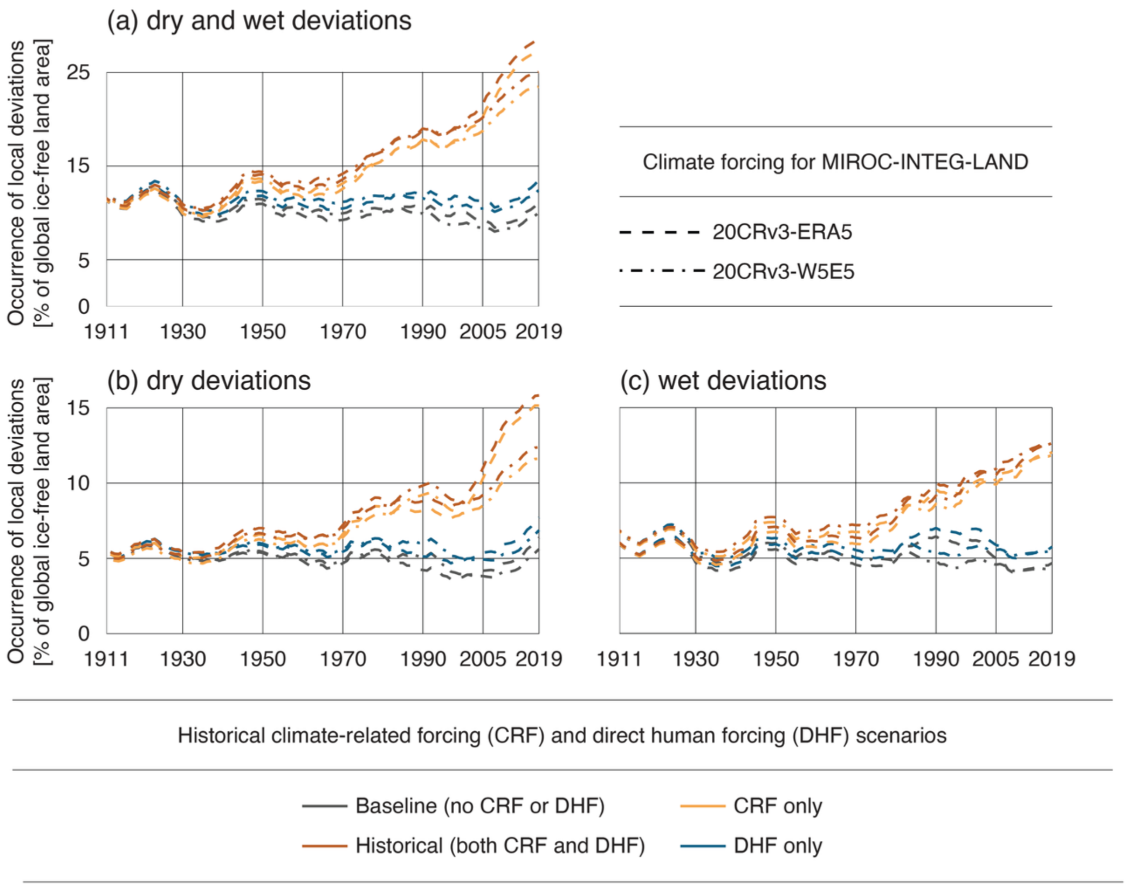

Supplementary Figure 2. Global occurrence of dry and wet local soil moisture deviations under different climate-related forcing (CRF) and direct human forcing (DHF) scenarios, separately for two MIROC-INTEG-LAND simulations using two different CRF data sets. The global occurrence of local soil moisture deviations is measured by the percentage share of global ice-free land area with local deviations, for dry and wet deviations (a), dry deviations (b), and wet deviations (c). Shown is the annual percentage, which is computed as an average of monthly percentages (Methods). Time series of the occurrence of local deviations are smoothed with a 10-year moving (trailing) mean over the annual percentage.

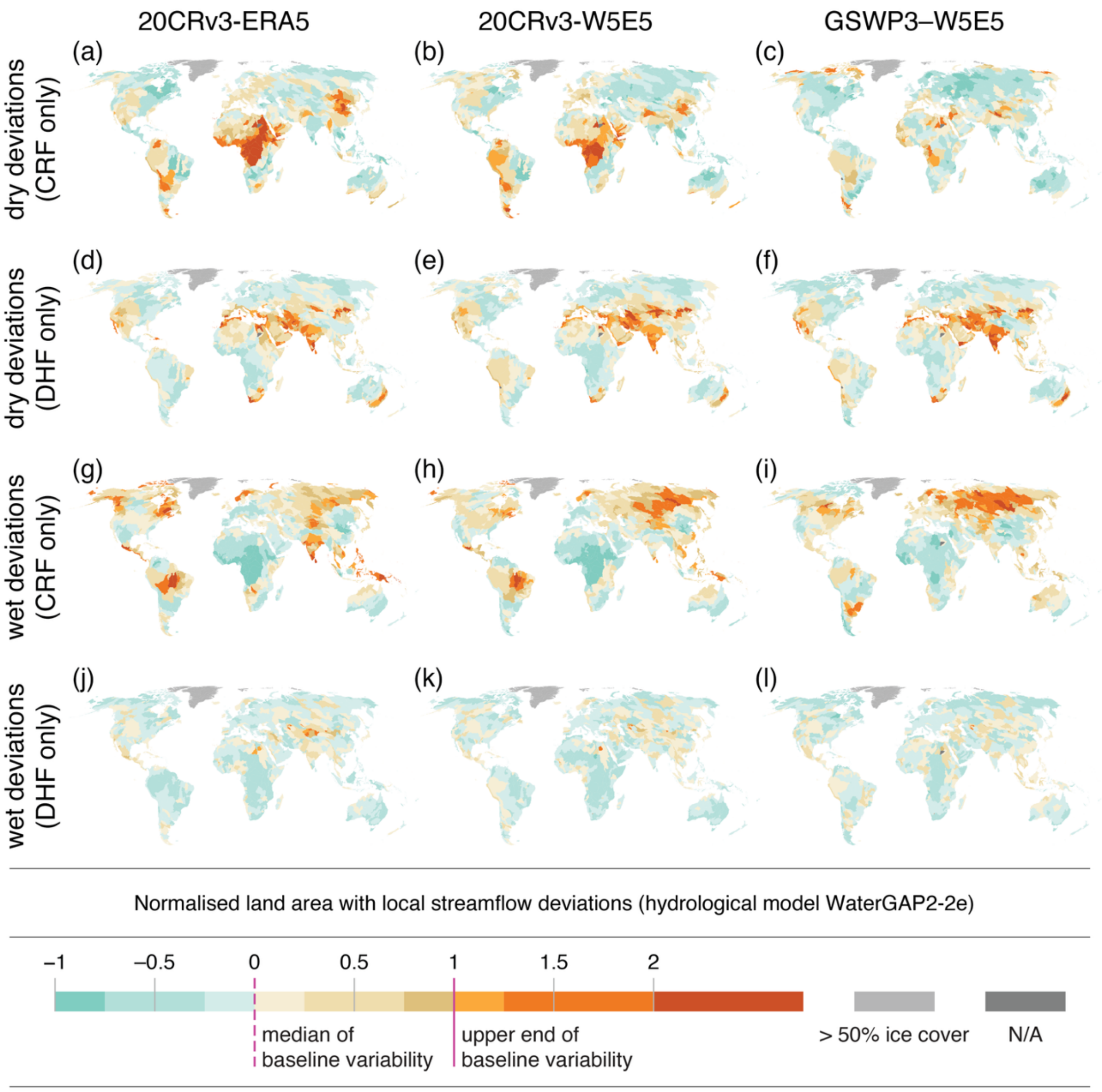

Supplementary Figure 3. Regional occurrence of dry and wet local streamflow deviations under different climate-related forcing (CRF) and direct human forcing (DHF) scenarios, separately for three WaterGAP2-2e simulations using three different CRF data sets. The regional occurrence of local deviations is measured by the normalised percentage share of regional ice-free land area with local deviations, for dry deviations (a–f) and wet deviations (g–l). Shown is the 30-year mean (1990–2019) regional deviation occurrence, taken from annual values to account for interannual variability (Fig. 3, Methods). Regions for which both the median and upper end of baseline variability equal to zero for the particular scenario and CRF data set are marked as non-applicable (N/A). The regions analysed here depict basins delineated by the HydroBASINS data set[9] level 4 (n = 1,280, mean area 103,000 km^2; median area 60,000 km^2).

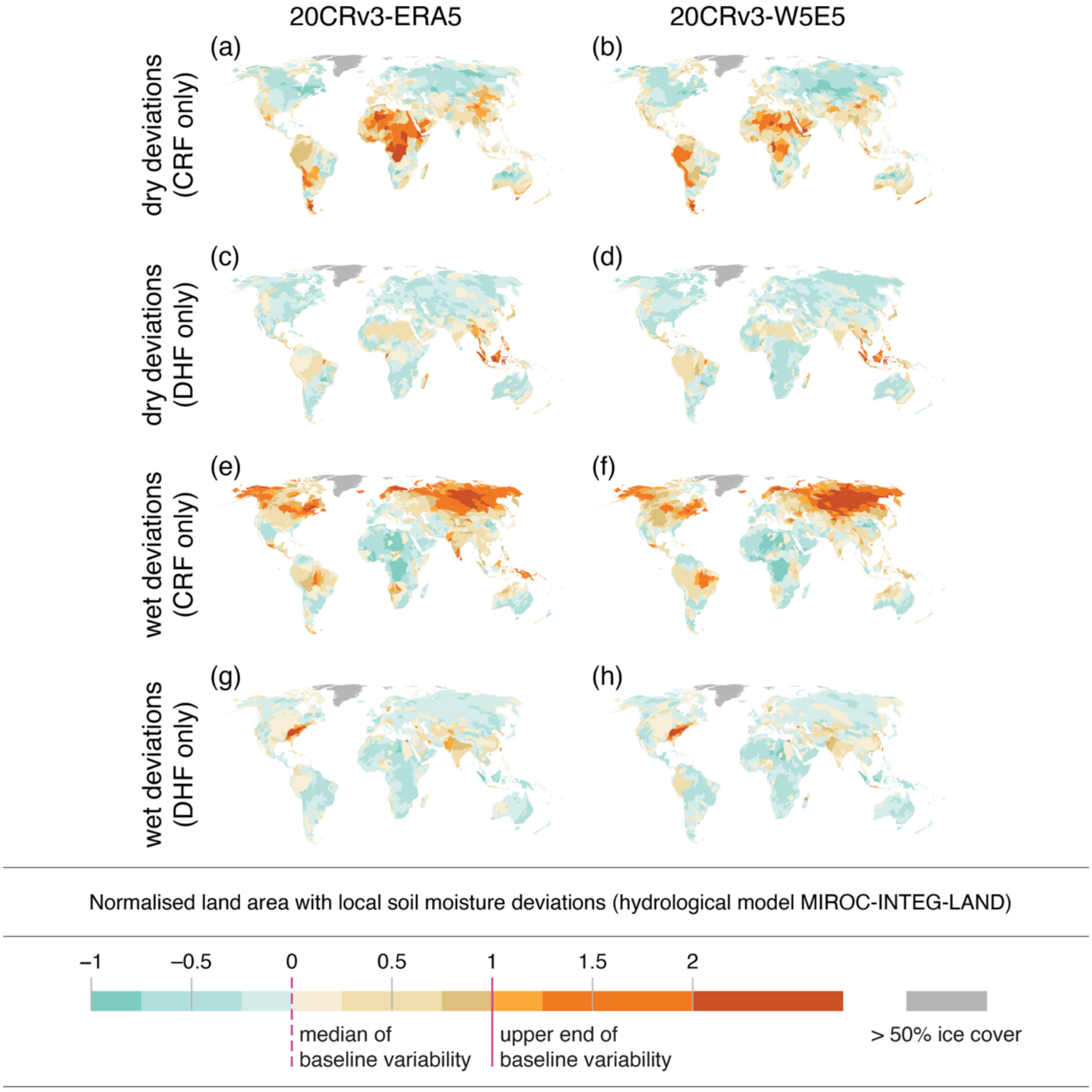

Supplementary Figure 4. Regional occurrence of dry and wet local soil moisture deviations under different climate-related forcing (CRF) and direct human forcing (DHF) scenarios, separately for two MIROC-INTEG-LAND simulations using two different CRF data sets. The regional occurrence of local deviations is measured by the normalised percentage share of regional ice-free land area with local deviations, for dry deviations (a–d) and wet deviations (e–h). Shown is the 30-year mean (1990–2019) regional deviation occurrence, taken from annual values to account for interannual variability (Fig. 3, Methods). The regions analysed here depict basins delineated by the HydroBASINS data set[9] level 4 (n = 1,280, mean area 103,000 km^2; median area 60,000 km^2).

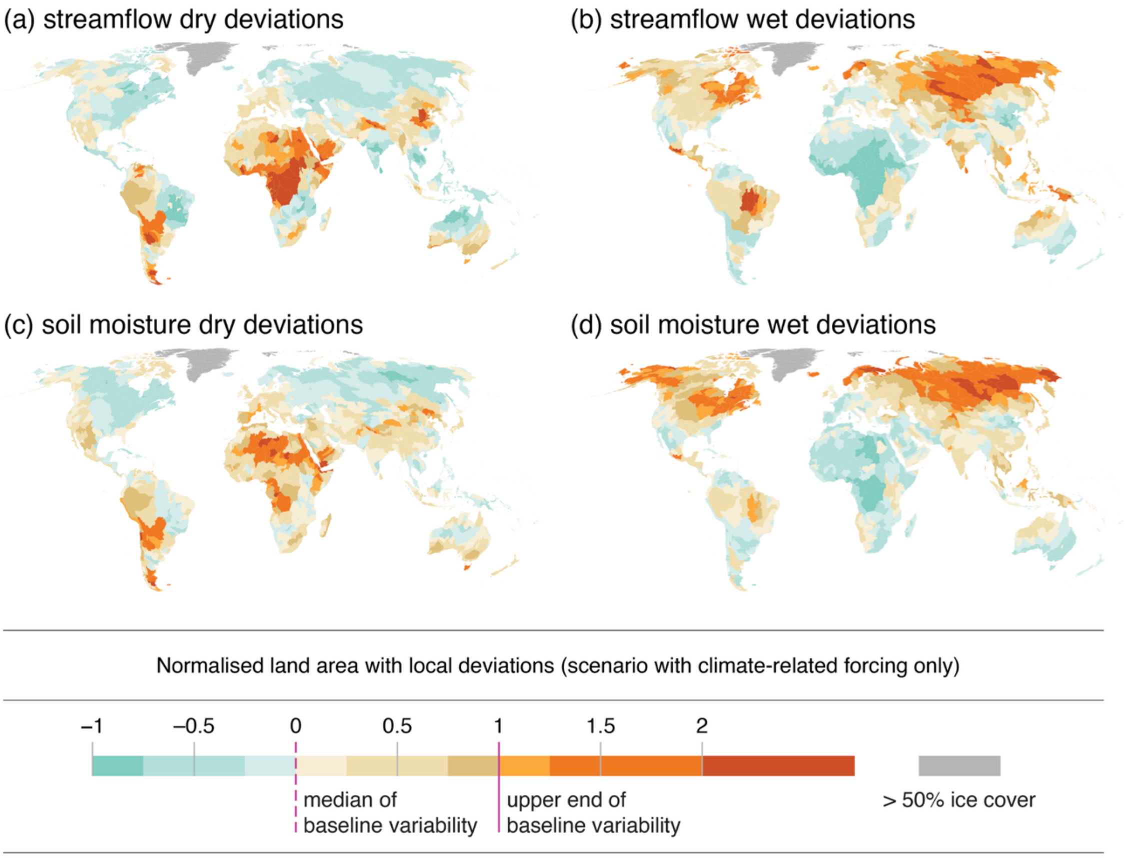

Supplementary Figure 5. Regional occurrence of dry and wet local deviations for a simulation scenario consisting of climate-related forcing only. The regional occurrence of local deviations is measured by the normalised percentage share of regional ice-free land area with local deviations, for dry streamflow deviations (a), wet streamflow deviations (b), dry soil moisture deviations (c), and wet soil moisture deviations (d). Shown is the 30-year mean (1990–2019) regional deviation occurrence, taken from annual ensemble medians to account for interannual variability (Fig. 3, Methods). The regions analysed here depict basins delineated by the HydroBASINS data set[9] level 4 (n = 1,280, mean area 103,000 km^2; median area 60,000 km^2).

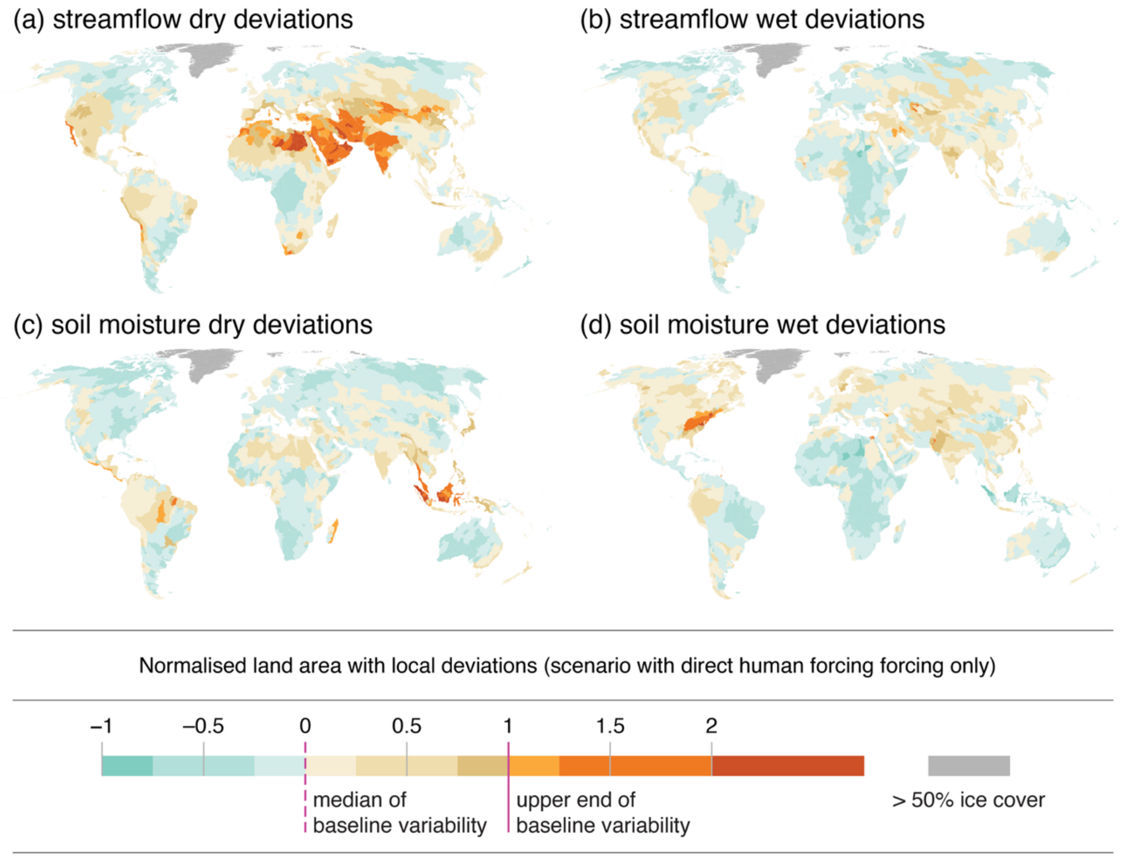

Supplementary Figure 6. Regional occurrence of dry and wet local deviations for a simulation scenario consisting of direct human forcing only. The regional occurrence of local deviations is measured by the normalised percentage share of regional ice-free land area with local deviations, for dry streamflow deviations (a), wet streamflow deviations (b), dry soil moisture deviations (c), and wet soil moisture deviations (d). Shown is the 30-year mean (1990–2019) regional deviation occurrence, taken from annual ensemble medians to account for interannual variability (Fig. 3, Methods). The regions analysed here depict basins delineated by the HydroBASINS data set[9] level 4 (n = 1,280, mean area 103,000 km^2; median area 60,000 km^2).

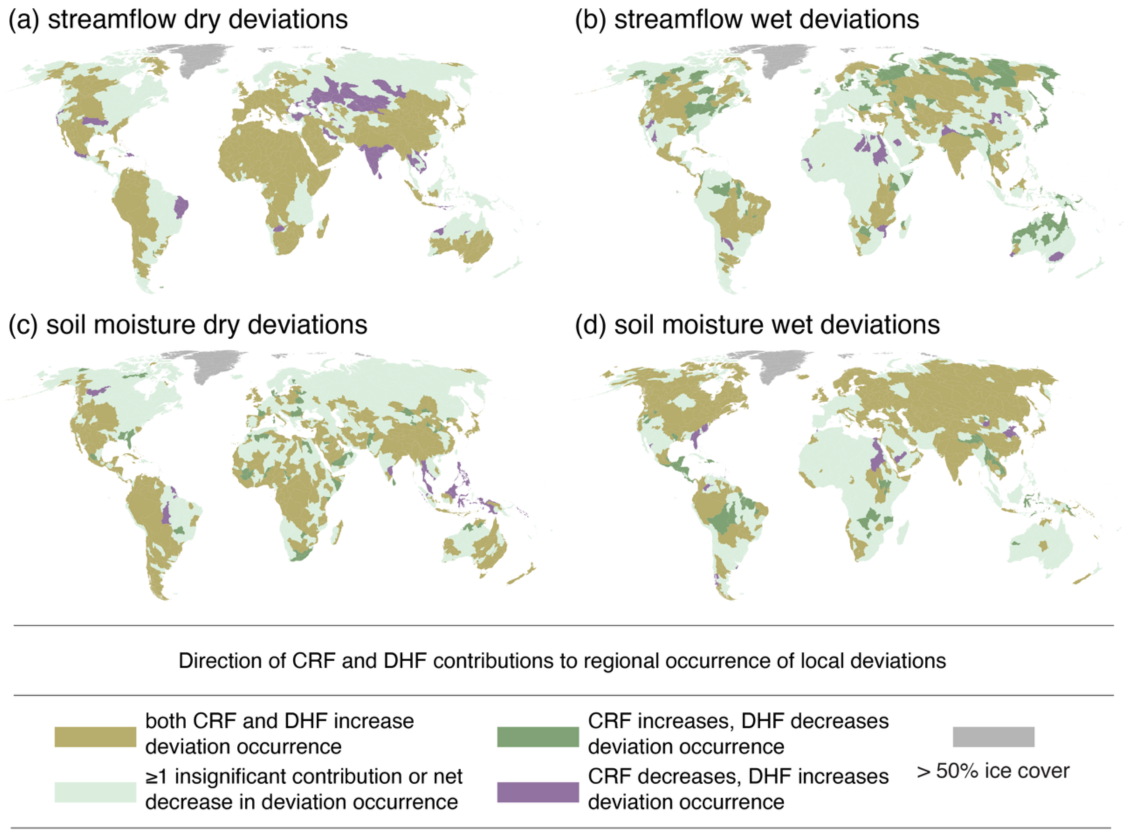

Supplementary Figure 7. Directionality of climate-related forcing (CRF) and direct human forcing (DHF) contributions to increasing regional occurrence of local deviations. The CRF and DHF contributions are based on comparison between each scenario against the baseline scenario (Fig. 3b, Methods), for dry streamflow deviations (a), wet streamflow deviations (b), dry soil moisture deviations (c), and wet soil moisture deviations (d). Before assessing contributions, 30-year means (1990–2019) of regional deviation occurrence are taken from annual ensemble medians to account for interannual variability. Scenario contributions are tested for statistical significance using the Wilcoxon signed rank sum test (two-tailed test, significance level $p = 0.05$, $n = 30$). The directionality of CRF and DHF contributions is assigned for those regions in which both CRF and DHF contributions are statistically significant (Fig. 5), and net deviation occurrence (Fig. 4) increases significantly. If either of these contributions is not significant or if net deviation occurrence does not increase significantly, the CRF and DHF contribution directionality is not assigned. The regions analysed here depict basins delineated by the HydroBASINS data set[9] level 4 ($n = 1{,}280$, mean area 103,000 km^2; median area 60,000 km^2).

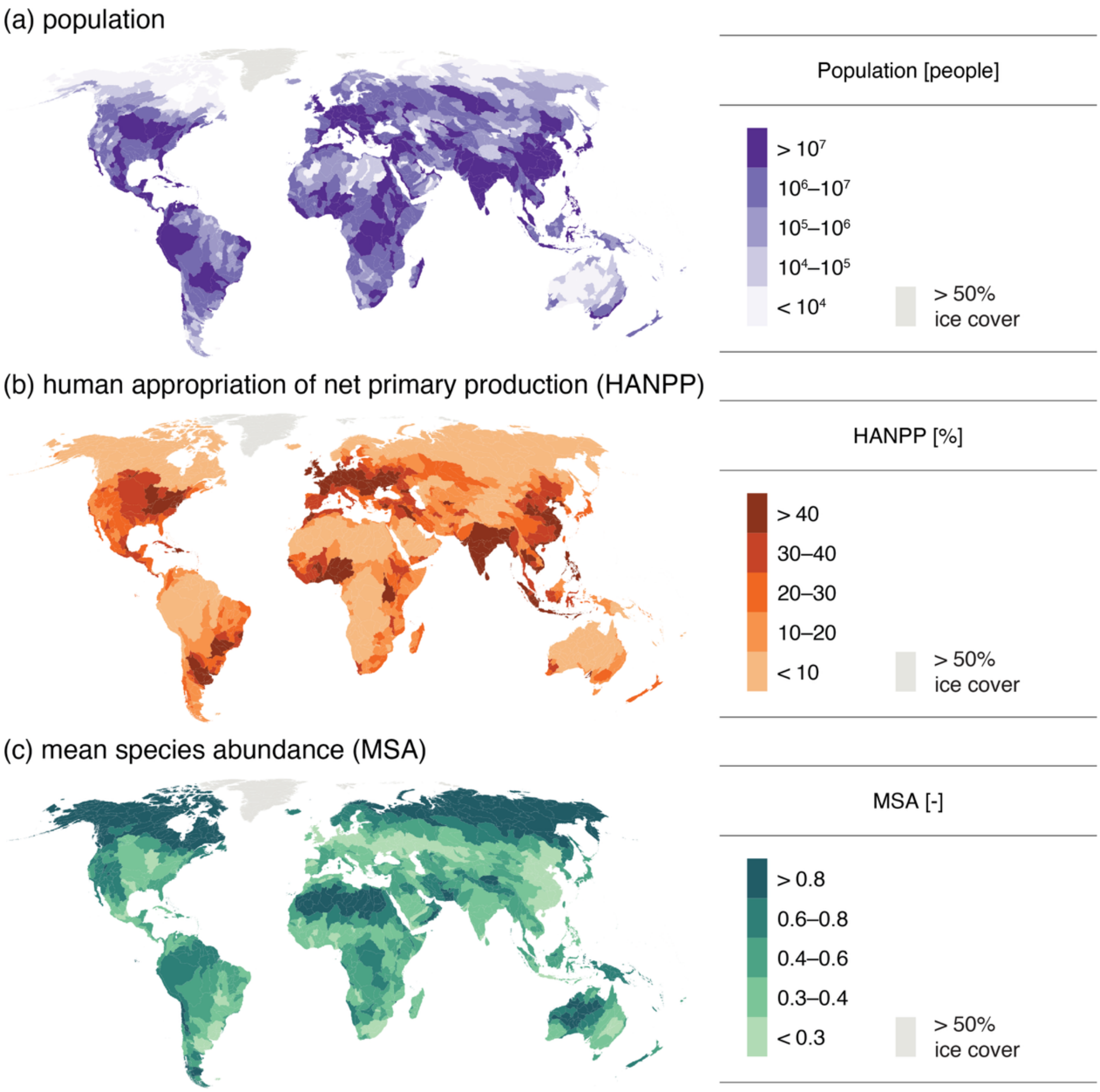

Supplementary Figure 8. Regionally aggregated auxiliary variables. The three auxiliary variables consist of population count[10] (a), human appropriation of net primary production[11] (HANPP) (b), and mean species abundance[12] (MSA) (c). Within each analysed HydroBASINS data set[9] level 4 catchment (n = 1,268), population count is aggregated with a zonal sum, whereas HANPP and MSA are aggregated with grid cell area weighted zonal averages (Methods). All operations consider only the grid cell fractions that are within the region boundaries[13] and weight the regional sum or average accordingly.

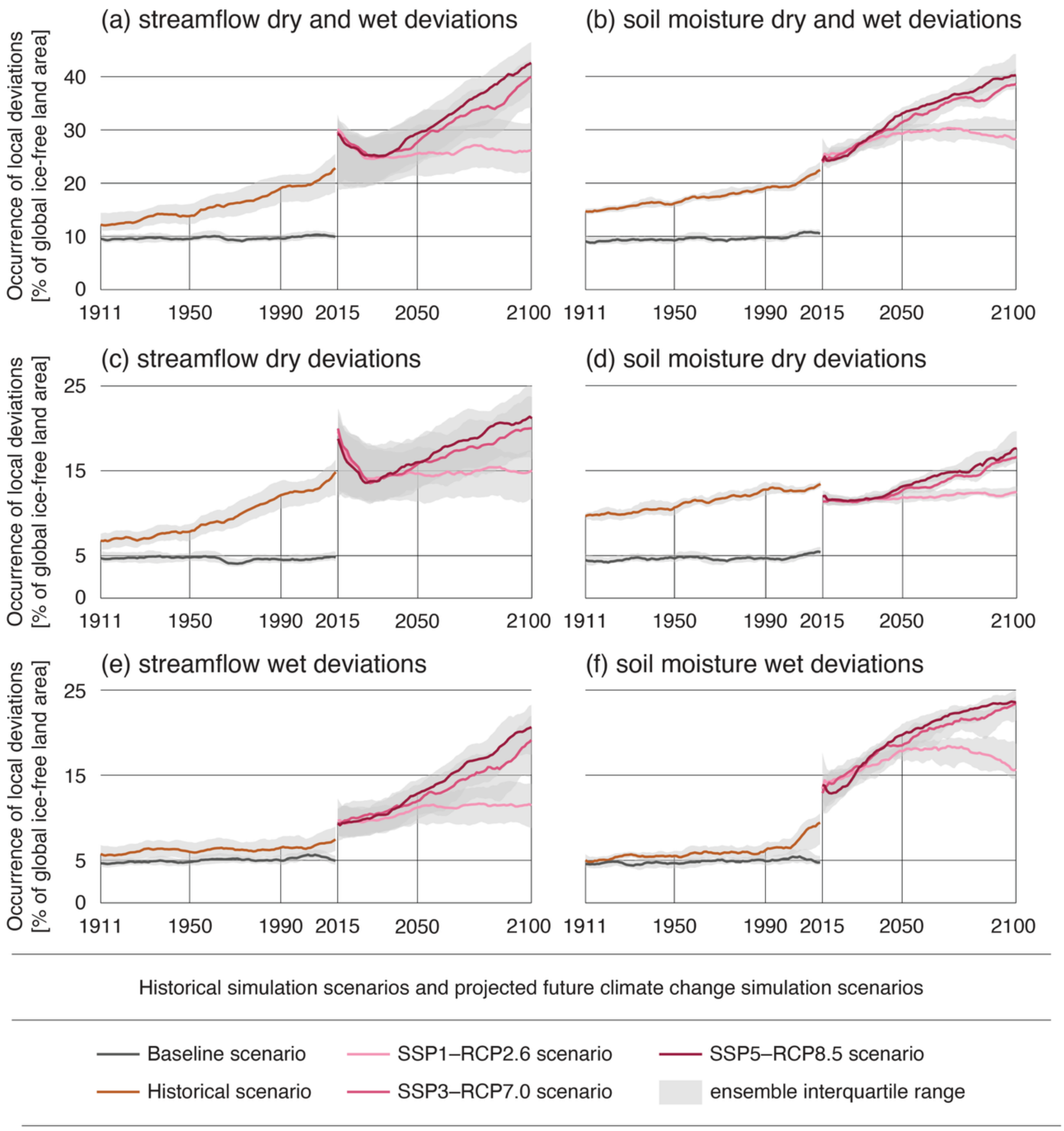

Supplementary Figure 9. Global occurrence of dry and wet local deviations under baseline, historical, and projected future climate change scenarios. The global occurrence of local deviations is measured by the percentage share of global ice-free land area with local deviations, for dry and wet streamflow deviations (a), dry and wet soil moisture deviations (b), dry streamflow deviations (c), dry soil moisture deviations (d), wet streamflow deviations (e), and wet soil moisture deviations (f). Hydrological simulations underlying global deviation occurrence in panels a–f are sourced from Inter-Sectoral Impact Model Intercomparison Project simulation round 3b experiments[2], which re-establishes all scenarios including the baseline scenario (Supplementary Text). Years prior to 1911 are excluded from panels a–f, although used in determining the occurrence of local deviations (Supplementary Text). Shown is the ensemble median of annual percentage, which is computed as an average of monthly percentages. Time series of the occurrence of local deviations and limits of the ensemble interquartile range (IQR) are smoothed with a 10-year moving (trailing) mean over the ensemble median and ensemble IQR limits, respectively.

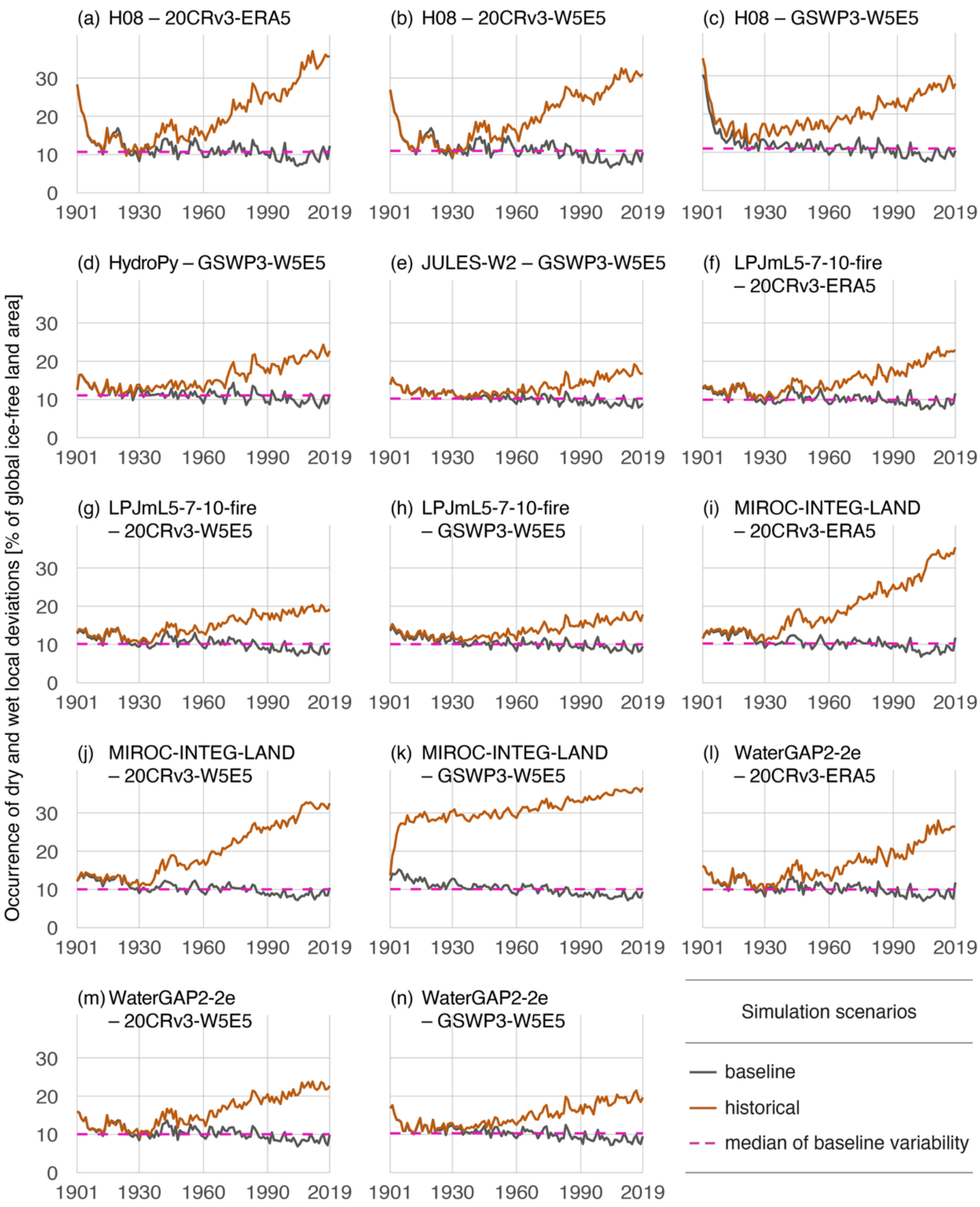

Supplementary Figure 10. Global occurrence of dry and wet local streamflow deviations under baseline and historical scenarios, for each hydrological model ensemble member separately. The global occurrence of local deviations is measured by the percentage share of global ice-free land area with local streamflow deviations, for dry and wet streamflow deviations and for each ensemble member (a–n) (Supplementary Table 2). Shown is the annual percentage, which is computed as an average of monthly percentages (Methods). Although excluded from determining the median of baseline variability, deviation occurrence during years 1901–1910 is shown here to illustrate potential traces of model spinup during the first years of each simulation (Methods).

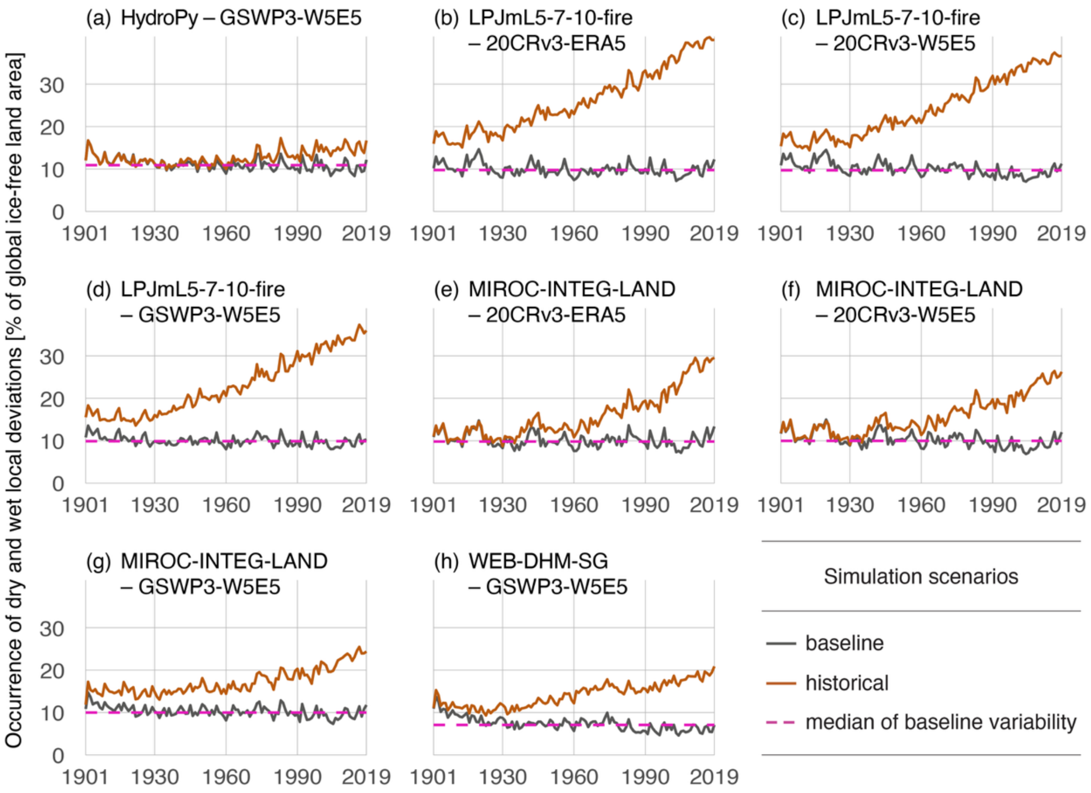

Supplementary Figure 11. Global occurrence of dry and wet local soil moisture deviations under baseline and historical scenarios, for each hydrological model ensemble member separately. The global occurrence of local deviations is measured by the percentage share of global ice-free land area with local soil moisture deviations, for dry and wet soil moisture deviations and for each ensemble member (a–h) (Supplementary Table 2). Shown is the annual percentage, which is computed as an average of monthly percentages (Methods). Although excluded from determining the median of baseline variability, deviation occurrence during years 1901–1910 is shown here to illustrate potential traces of model spinup during the first years of each simulation (Methods).

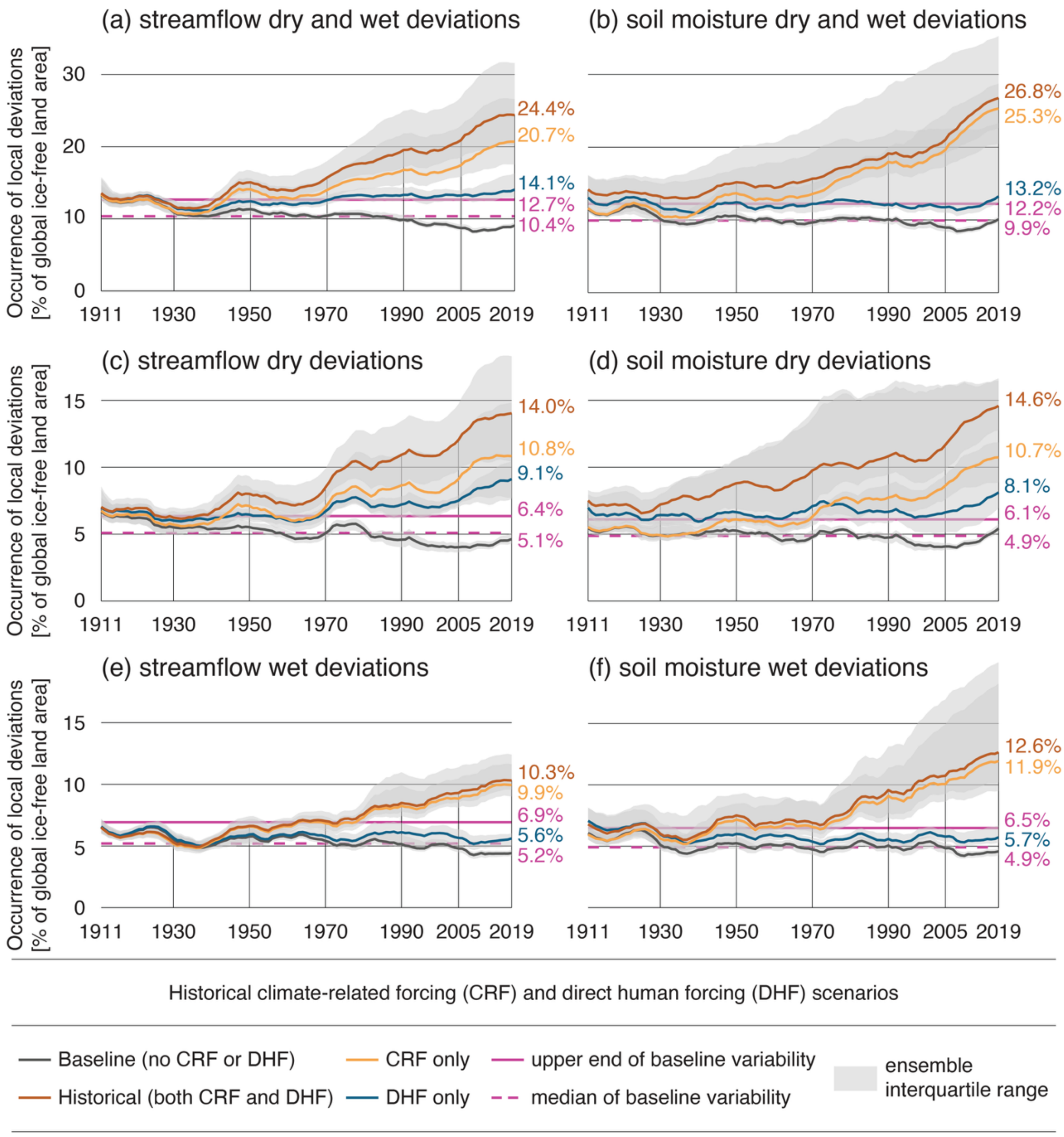

Supplementary Figure 12. Global occurrence of dry and wet local deviations under different climate-related forcing (CRF) and direct human forcing (DHF) scenarios, including otherwise excluded ensemble members. The global occurrence of local deviations is measured by the percentage share of global ice-free land area with local deviations, for dry and wet streamflow deviations (a), dry and wet soil moisture deviations (b), dry streamflow deviations (c), dry soil moisture deviations (d), wet streamflow deviations (e), and wet soil moisture deviations (f). Shown is the ensemble median of annual percentage, which is computed as an average of monthly percentages (Methods). Time series of the occurrence of local deviations and limits of the ensemble interquartile range (IQR) are smoothed with a 10-year moving (trailing) mean over the ensemble median and ensemble IQR limits, respectively. When including otherwise excluded ensemble members, the total streamflow data ensemble size becomes n = 14, and the total soil moisture data ensemble size becomes n = 8 (Methods, Supplementary Table 2).

Supplementary Tables

Supplementary Table 1. Group sizes for percentile rank bins synthesising across climate-related forcing (CRF) and direct human forcing (DHF) contributions on regional freshwater deviation occurrence. Global percentile ranks are assigned for scenario contributions in each of the four cases of regional deviation occurrence (streamflow and soil moisture, dry and wet), and the groups (Fig. 6) are created by binning the median of these four ranks for CRF and DHF scenarios separately (Methods). The N/A percentile rank bin captures those 12 regions that did not overlap spatially with all three auxiliary data variables analysed in Fig. 6.

percentile rank bin	group size (CRF scenario)	group size (DHF scenario)
[0, 0.2)	3	35
[0.2, 0.4)	150	416
[0.4, 0.6)	880	400
[0.6, 0.8)	222	302
[0.8, 1]	13	115
N/A	12	12

Supplementary Table 2. Ensemble members comprising the global hydrological model (GHM) ensemble (combinations of GHMs and climate-related forcing (CRF) data sets) used in this study. All ensemble members that were available in the Inter-Sectoral Impact Model Intercomparison Project simulation round 3a repository[1,14] with simulations for all desired scenarios were initially selected, and the selection was narrowed down by discarding those ensemble members that were found to mismatch between baseline and historical scenarios in the beginning of the simulation period (Methods).

		CRF data sets for which simulations are available		
variable	GHM	20CRv3-ERA5	20CRv3-W5E5	GSWP3-W5E5
streamflow	H08	x (included)	x (included)	x (excluded)
streamflow	HydroPy	(not available)	(not available)	x (included)
streamflow	JULES-W2	(not available)	(not available)	x (included)
streamflow	LPJmL5-7-10-fire	x (included)	x (included)	x (included)
streamflow	MIROC-INTEG-LAND	x (included)	x (included)	x (excluded)
streamflow	WaterGAP2-2e	x (included)	x (included)	x (included)
soil moisture	HydroPy	(not available)	(not available)	x (included)
soil moisture	LPJmL5-7-10-fire	x (excluded)	x (excluded)	x (excluded)
soil moisture	MIROC-INTEG-LAND	x (included)	x (included)	x (excluded)
soil moisture	WEB-DHM-SG	(not available)	(not available)	x (included)

available (included in main results)
available (excluded from main results)
not available

Supplementary References

1. Frieler, K. *et al.* Scenario setup and forcing data for impact model evaluation and impact attribution within the third round of the Inter-Sectoral Model Intercomparison Project (ISIMIP3a). *Geosci. Model Dev.* **17**, 1–51 (2024).
2. Gosling, S. N. *et al.* ISIMIP3b Simulation Data from the Global Water Sector. ISIMIP Repository https://doi.org/10.48364/ISIMIP.230418.7 (2025).
3. ISIMIP. ISIMIP3b protocol for water_global. https://protocol.isimip.org/#/ISIMIP3b/water_global (2025).
4. Hanasaki, N., Yoshikawa, S., Pokhrel, Y. & Kanae, S. A global hydrological simulation to specify the sources of water used by humans. *Hydrol. Earth Syst. Sci.* **22**, 789–817 (2018).
5. Yokohata, T. *et al.* MIROC-INTEG-LAND version 1: a global biogeochemical land surface model with human water management, crop growth, and land-use change. *Geosci. Model Dev.* **13**, 4713–4747 (2020).
6. Müller Schmied, H. *et al.* The global water resources and use model WaterGAP v2.2e: description and evaluation of modifications and new features. *Geosci. Model Dev.* **17**, 8817–8852 (2024).
7. Lange, S. Trend-preserving bias adjustment and statistical downscaling with ISIMIP3BASD (v1.0). *Geosci. Model Dev.* **12**, 3055–3070 (2019).
8. Porkka, M. *et al.* Notable shifts beyond pre-industrial streamflow and soil moisture conditions transgress the planetary boundary for freshwater change. *Nat. Water* **2**, 262–273 (2024).
9. Lehner, B. & Grill, G. Global river hydrography and network routing: baseline data and new approaches to study the world's large river systems. *Hydrol. Process.* **27**, 2171–2186 (2013).
10. Klein Goldewijk, K. HYDE data for the Global Carbon Budget (2025). Utrecht University https://doi.org/10.24416/UU01-F45D44 (2025).
11. Kastner, T. *et al.* Land use intensification increasingly drives the spatiotemporal patterns of the global human appropriation of net primary production in the last century. *Glob. Change Biol.* **28**, 307–322 (2022).
12. Schipper, A. M. *et al.* Projecting terrestrial biodiversity intactness with GLOBIO 4. *Glob. Change Biol.* **26**, 760–771 (2020).
13. Baston, D. exactextractr: Fast Extraction from Raster Datasets using Polygons. (2023).
14. Gosling, S. N. *et al.* ISIMIP3a Simulation Data from the Global Water Sector. ISIMIP Repository https://doi.org/10.48364/ISIMIP.398165.10 (2025).
