# Supplementary material for: Regionally divergent drivers behind transgressions of the freshwater change planetary boundary
Source: Nat Commun. 2026 May 13;17:5132. doi: 10.1038/s41467-026-73051-x (PMC13250156; doi:10.1038/s41467-026-73051-x)
Supplement: Supplementary file 2 — Reporting Summary [file 41467_2026_73051_MOESM2_ESM.pdf]

Corresponding author(s): Vili Virkki
Last updated by author(s): Apr 27, 2026

Reporting Summary

Nature Portfolio wishes to improve the reproducibility of the work that we publish. This form provides structure for consistency and transparency in reporting. For further information on Nature Portfolio policies, see our Editorial Policies and the Editorial Policy Checklist.

Statistics

For all statistical analyses, confirm that the following items are present in the figure legend, table legend, main text, or Methods section.

n/a	Confirmed	
☒	☐	The exact sample size (*n*) for each experimental group/condition, given as a discrete number and unit of measurement
☒	☐	A statement on whether measurements were taken from distinct samples or whether the same sample was measured repeatedly
☐	☒	The statistical test(s) used AND whether they are one- or two-sided<br>*Only common tests should be described solely by name; describe more complex techniques in the Methods section.*
☒	☐	A description of all covariates tested
☒	☐	A description of any assumptions or corrections, such as tests of normality and adjustment for multiple comparisons
☒	☐	A full description of the statistical parameters including central tendency (e.g. means) or other basic estimates (e.g. regression coefficient) AND variation (e.g. standard deviation) or associated estimates of uncertainty (e.g. confidence intervals)
☒	☐	For null hypothesis testing, the test statistic (e.g. *F*, *t*, *r*) with confidence intervals, effect sizes, degrees of freedom and *P* value noted<br>*Give P values as exact values whenever suitable.*
☒	☐	For Bayesian analysis, information on the choice of priors and Markov chain Monte Carlo settings
☒	☐	For hierarchical and complex designs, identification of the appropriate level for tests and full reporting of outcomes
☒	☐	Estimates of effect sizes (e.g. Cohen's *d*, Pearson's *r*), indicating how they were calculated

Our web collection on statistics for biologists contains articles on many of the points above.

Software and code

Policy information about availability of computer code

Data collection	All data collection and analyses were conducted with R version 4.5.2. The code used in producing the results shown will be made publicly available upon publication of the article, as described in the Code availability statement.
Data analysis	All data collection and analyses were conducted with R version 4.5.2. The code used in producing the results shown will be made publicly available upon publication of the article, as described in the Code availability statement.

For manuscripts utilizing custom algorithms or software that are central to the research but not yet described in published literature, software must be made available to editors and reviewers. We strongly encourage code deposition in a community repository (e.g. GitHub). See the Nature Portfolio guidelines for submitting code & software for further information.

Data

Policy information about availability of data

All manuscripts must include a data availability statement. This statement should provide the following information, where applicable:

- Accession codes, unique identifiers, or web links for publicly available datasets
- A description of any restrictions on data availability
- For clinical datasets or third party data, please ensure that the statement adheres to our policy

All data used in this study were gathered from openly available sources, which are appropriately cited in Methods. Output data produced by the analysis will be deposited in a public database upon publication of the article, as described in the Data availability statement.

Research involving human participants, their data, or biological material

Policy information about studies with human participants or human data. See also policy information about sex, gender (identity/presentation), and sexual orientation and race, ethnicity and racism.

Reporting on sex and gender	no information on sex or gender was collected
Reporting on race, ethnicity, or other socially relevant groupings	no information on race, ethnicity or other socially relevant groupings was collected
Population characteristics	no human research participants were involved
Recruitment	no human research participants were involved
Ethics oversight	ethics oversight not required because no human research participants were involved

Note that full information on the approval of the study protocol must also be provided in the manuscript.

Field-specific reporting

Please select the one below that is the best fit for your research. If you are not sure, read the appropriate sections before making your selection.

☐ Life sciences ☐ Behavioural & social sciences ☒ Ecological, evolutionary & environmental sciences

For a reference copy of the document with all sections, see nature.com/documents/nr-reporting-summary-flat.pdf

Ecological, evolutionary & environmental sciences study design

All studies must disclose on these points even when the disclosure is negative.

Study description	The study analyses global hydrological simulations to assess change in the freshwater cycle.
Research sample	Most data used in the study are simulated hydrological data, which are available in the ISIMIP repository (https://data.isimip.org). Other, auxiliary data sets consist of openly available global geospatial data. All data sources used in this study are appropriately cited in Methods.
Sampling strategy	No sampling was done for the data
Data collection	No primary data collection was done by the authors; all data were existing data sets
Timing and spatial scale	No primary data collection was done by the authors; all data were existing data sets
Data exclusions	No data were excluded from the analysis
Reproducibility	No experimental settings based on primary data were included in the study. The code used in producing the results shown will be made publicly available upon publication of the article, as described in the Code availability statement.
Randomization	No experimental settings based on primary data requiring randomization were included in the study
Blinding	No experimental settings based on primary data requiring blinding were included in the study

Did the study involve field work? ☐ Yes ☒ No

Reporting for specific materials, systems and methods

We require information from authors about some types of materials, experimental systems and methods used in many studies. Here, indicate whether each material, system or method listed is relevant to your study. If you are not sure if a list item applies to your research, read the appropriate section before selecting a response.

Materials & experimental systems

n/a	Involved in the study
☒	☐ Antibodies
☒	☐ Eukaryotic cell lines
☒	☐ Palaeontology and archaeology
☒	☐ Animals and other organisms
☒	☐ Clinical data
☒	☐ Dual use research of concern
☒	☐ Plants

Methods

n/a	Involved in the study
☒	☐ ChIP-seq
☒	☐ Flow cytometry
☒	☐ MRI-based neuroimaging

Plants

Seed stocks	n/a
Novel plant genotypes	n/a
Authentication	n/a
