# Supplementary material for: Regionally divergent drivers behind transgressions of the freshwater change planetary boundary
Source: Nat Commun. 2026 May 13;17:5132. doi: 10.1038/s41467-026-73051-x (PMC13250156; doi:10.1038/s41467-026-73051-x)
Supplement: Supplementary file 3 — Transparent Peer Review file [file 41467_2026_73051_MOESM3_ESM.pdf]

nature portfolio

Peer Review File

Regionally divergent drivers behind transgressions of the freshwater change planetary boundary

Corresponding Author: Dr Vili Virkki

This file contains all reviewer reports in order by version, followed by all author rebuttals in order by version.

Version 0:

Reviewer comments:

Reviewer #1

(Remarks to the Author)
Review of ‘ Regionally divergent drivers behind transgressions of the freshwater change planetary boundary’ by Vili Virkki and others.

The authors expand on the work by Porkka et al. (2024) by extending the PB-FW analysis till 2019 and decompose the global and regional drivers of streamflow and soil moisture deviations by using the model outputs from the latest ISIMIP simulation round. The manuscript is well written, but in our opinion some points need to be clarified/improved to increase readability and before we would recommend publication.

General comments:

- My largest concern with the manuscript in its current form is that the relevance and implications of the research are unclear (the ‘why’ is missing). In the abstract is not indicated which scientific/societal question is exactly addressed by this paper and why this is needed to be answered. It mainly feels like an incremental research, that builds on previous studies by the same authors by expanding the time line and decomposition of the main drivers of change in planetary boundary transgression. But what are the implications of the results in this study? What does a regional decomposition mean for preventing further transgressions for example? I am not yet sure that the paper provides enough scientific advances to justify publication in a Nature journal, but I think the manuscript would benefit greatly from some more societal context, some elaboration on the why and discussion on implications.
- I miss an underpinning of why the ‘variance in the occurrence of local deviations under the baseline scenario’ is set as the planetary boundary for green and blue freshwater? Could the authors indicate what it exactly means and why this value is considered a ‘boundary’? Do I understand this correctly as ‘the percentage of global area that is at the same time experiencing a drought or a flood’?
- It is not clear why some sort of hypothesis testing (at both global and regional level) is not carried out to identify whether the scenario contributions are significant, especially for DHF contributions which look quite similar to the baseline. Such tests seem to have been carried out for trend significance, but no information is provided for attribution.

- Discussion regarding the regional trends and drivers and how they compare with existing regional studies can be improved. Linking this discussion to possible regional implications and recommendations for specific regional research would add value to the manuscript.
Specific comments:
1) L14: The first line of abstract seems to be at odds with L325-327.
2) L102: Definition of persistently transgressed is not clear. Looks like this comes from Porkka et al. (2024), so could be a good idea to reintroduce in this manuscript. Also, some explanation on why this persistent transgression occurs after 1940 would be beneficial to the reader.
3) L106: The phrase “indicates … elevated freshwater related risks in the earth system” may be better suited for discussion section.
4) Fig.2: Plotting the upper end of variability would be useful as this is also used to define the upper limit.
5) L161: It is not clear why hydroBASINS level 4 is chosen in particular for regional analysis
6) Fig. 3 and methods: It is not clear why 1990-2019 is the period chosen for regional analysis
7) L188-190: These lines are difficult to follow

8) Synthesis: It is not clear why the median of four percentiles is used to compare relative contribution. Why not aggregate the (area of) deviations first and then rank the percentiles to assess the overall contribution?
9) L282-286: Since this paragraph is mostly “exploratory”, as in it intends to visually identify some association between scenario contributions and the three auxiliary variables (presumably to guide further research), it might be a good idea to also qualify these sentences with words such as “likely”, “appears to”, etc. (also for related parts in discussion and conclusion)
10) L357-360: It’s not clear what exactly is meant by “…takes a step forward” and “offers an actionable use case”. Do these relate to Fig. 6c-e?
11) L381: It may be beneficial for the readers to note that the baseline conditions in this manuscript also differ from Porkka et al (2024) where pre-industrial simulations are used to define the baseline as opposed to counterfactual simulations here.
12) L418: It’s not clear why this mismatch occurs and why this justifies removing ensembles from analysis in the main text, especially since some initial years are discarded anyway owing to model spinup (L456).
13) L484: It is not clear why monthly means is chosen to estimate annual deviation and what its implications are.

References
Porkka, M., Virkki, V., Wang-Erlandsson, L., Gerten, D., Gleeson, T., Mohan, C., Fetzer, I., Jaramillo, F., Staal, A., Te Wierik, S., Tobian, A., Van Der Ent, R., Döll, P., Flörke, M., Gosling, S.N., Hanasaki, N., Satoh, Y., Müller Schmied, H., Wanders, N., Famiglietti, J.S., Rockström, J., Kummu, M., 2024. Notable shifts beyond pre-industrial streamflow and soil moisture conditions transgress the planetary boundary for freshwater change. Nat Water 2, 262–273. https://doi.org/10.1038/s44221-024-00208-7

(Remarks on code availability)

Reviewer #2

(Remarks to the Author)
I co-reviewed this manuscript with one of the reviewers who provided the listed reports. This is part of the Nature Communications initiative to facilitate training in peer review and to provide appropriate recognition for Early Career Researchers who co-review manuscripts.

(Remarks on code availability)

Reviewer #3

(Remarks to the Author)
Review of “Regionally divergent drivers behind transgressions of the freshwater change planetary boundary” by Virkki et al.

Recommendation: Major revisions

This study examines the planetary boundaries of freshwater change. It uses the latest version of the corresponding ISIMIP simulations. Understanding the drivers of transgressing planetary boundaries is an important and timely topic and this study makes a worthwhile contribution to the literature. The manuscript is well written but I have several comments which need to be addressed. One particular weakness is that in my opinion the analysis methods are not described in any kind of detail.

1) Line 30: Volatility can potentially also contribute to this. Extreme precip following a dry period, water might run off on the surface instead of sinking into ground.
E.g.
Nordling, K., Fahrenbach, N. L. S., and Samset, B. H.: Climate variability can outweigh the influence of climate mean changes for extreme precipitation under global warming, Atmos. Chem. Phys., 25, 1659–1684, https://doi.org/10.5194/acp-25-1659-2025, 2025.
Franzke, C., 2022: Changing temporal volatility of precipitation extremes due to global warming. Int. J. Climatol., 42, 8971-8983.

2) Line 71: Why this particular range? Extreme water scarcity can occur when water reservoir levels drop to about 13% of capacity.; e.g Cape Town in 2018 which then also coined the term Day Zero Drought.

3) Line 80: “Maximally large”: It is disputable whether your ensemble is maximally large. You could perform your own additional simulations.

4) Line 105: Are those trends statistically significant? Do you compute linear trends? Do you take account of serial correlations?
In Fig. 1 it is visible that the mean and median are different most of the time. This suggests that the data are non-Gaussian. Has this been taken account of in the trend analysis?
More information is needed here.

5) I think the use of percentage points (pp) is somewhat non-standard. Why don’t you provide the relative change in %?

6) Line 243: Maybe you should discuss these regions in more detail.

7) Figure 6: This figure is hard to understand. In the legend you have the dark orange and blue colors but not the light colors. In the caption you talk about "shaded". I assume you mean the light colors. Maybe you should remove them if they play negligible roles.

8) Lines 278-280: This sentence needs more explanation and discussion.

9) Line 375: You need to explain the procedure. It is totally unclear what has been done.

10) Line 411-412: Is soil moisture data used also at monthly time scale?

11) Line 415: These are model data, why do they have missing/negative values? How does the data infilling affect the results? There are of course various ways you can do data infilling.

12) Line 461-463: Does this differ from Time of Emergence estimations?
E.g. Hawkins, E., and R. Sutton (2012), Time of emergence of climate signals, Geophys. Res. Lett., 39, L01702, doi:10.1029/2011GL050087.

13) Line 465-466: Most drought studies use running means to look at anomalous conditions. For computing SPI/SPEI you typically use a 3-month or up to 36/38 month running mean. Is this been done here too? If not, why not?

14) Eq. (1): To me the equation seems to provide negative values. You say it should be between 0 and 1.

15) Line 525: You could also look at nonlinear contributions following study by:
Ma, Q. and C. Franzke, 2021: The role of transient eddies and diabatic heating in the maintenance of European heat waves: a nonlinear stationary wave perspective. Climate Dyn., 56, 2983-3002.

16) Why do you use two different versions of the HYDE data base? Does this affect your results?

(Remarks on code availability)

Reviewer #4

(Remarks to the Author)
This manuscript provides a very interesting and relevant addition to the recent literature on the freshwater planetary boundary. Building on previous work by Porkka et al., the assessment of the PB-FW is extended to the last decades and deepened, particularly by providing regional attributions of climate change and direct human impacts on the two dimensions of the PB (green and blue water). The study is well-designed and the manuscript reads very well. I do have some suggestions and requests.

On a conceptual level, the PBs are by definition a global aggregate. Although I agree that regional differentiation is an important and interesting exercise, possible spatial differences thresholds are not considered. That is, for example, for some regions a 99th percentile of historical variability in soil moisture might have adverse systemic effects and for other ones it would be the 95th percentile. Although I would not advocate for a revised definition of the PB-FW itself, it can, however, reasonably be argued that downscaling the PB under the assumption that the globally aggregated 95th percentiles apply also at more local scales leaves out important local detail. If the authors agree, it would be good to see a more explicit discussion of this limitation.

The planetary boundaries can, of course, never be completely disentangled. For example, land cover change affects the climate, but both processes can and should still be considered separately in the PB framework. Indeed, the authors find that, globally, transgressions of the freshwater boundary are predominantly attributed to climate change. This is discussed in the paragraph starting in line 303, where this result is presented as a strength, because it underscores the interconnectedness of the Earth System. But could there also be negative implications of this result for the PB framework? Wouldn't it possibly undermine the validity of the PB-FW as a separate PB if transgressions are already captured by a (transgressed) climate PB? Here again, I do not argue for a fundamental revision of this PB, but a more critical reflection seems in place.

Lines 123-125: This can be established statistically rather than through simple eyeballing. Please provide a more objective way of reporting this important difference between dry and wet deviations.

Minor points:
155: This is about the past, so the past tense seems more appropriate.
292 and elsewhere: "The last 15 years" is not entirely accurate as the analysis ends in 2019.

(Remarks on code availability)

Version 1:

Reviewer comments:

Reviewer #1

(Remarks to the Author)
I would like to thank the authors for extensive answers to the reviewers comments. I can see the manuscript increased in clearity from answering to those comments.
My initial concerns, that are unrelated to the quality of the analysis and the writing, but more fundamental to the choice of these indicators to identify planetary boundaries for freshwater, remain.

Although the authors have now made the link to societal impact, and explained that the indicators can be used to support adaptation decisions, I still find these indicators a bit arbitrary. Argueing that the 'societies depend on a stable Earth system', seems to be a bit simple.

At the same time, I do not have a solution for this, as I think this issue is related to defining 'planetary boundaries' in general, also for the other elements of the planetary boundaries. Coming up with an indicator that is really meaningful and has clear implications when transgressed, is rather difficult. Still it is a very valuable framework to show the state of our Earth System and raise awareness of where it is heading.

(Remarks on code availability)

Reviewer #2

(Remarks to the Author)
I co-reviewed this manuscript with one of the reviewers who provided the listed reports. This is part of the Nature Communications initiative to facilitate training in peer review and to provide appropriate recognition for Early Career Researchers who co-review manuscripts.

(Remarks on code availability)

Reviewer #3

(Remarks to the Author)
The authors have addressed all my comments. I recommend to accept the manuscript for publication now.

(Remarks on code availability)

Reviewer #4

(Remarks to the Author)
I thank the authors for their careful and detailed replies and revisions and am happy to recommend publication of this work.

(Remarks on code availability)

Open Access This Peer Review File is licensed under a Creative Commons Attribution 4.0 International License, which permits use, sharing, adaptation, distribution and reproduction in any medium or format, as long as you give appropriate credit to the original author(s) and the source, provide a link to the Creative Commons license, and indicate if changes were made.
In cases where reviewers are anonymous, credit should be given to 'Anonymous Referee' and the source.
The images or other third party material in this Peer Review File are included in the article's Creative Commons license, unless indicated otherwise in a credit line to the material. If material is not included in the article's Creative Commons license and your intended use is not permitted by statutory regulation or exceeds the permitted use, you will need to obtain permission directly from the copyright holder.
To view a copy of this license, visit https://creativecommons.org/licenses/by/4.0/

Point-by-point response to the reviewers' comments of

Regionally divergent drivers behind transgressions of the freshwater change planetary boundary

Reviewer #1 (Remarks to the Author):

Review of ' Regionally divergent drivers behind transgressions of the freshwater change planetary boundary' by Vili Virkki and others.

The authors expand on the work by Porkka et al. (2024) by extending the PB-FW analysis till 2019 and decompose the global and regional drivers of streamflow and soil moisture deviations by using the model outputs from the latest ISIMIP simulation round. The manuscript is well written, but in our opinion some points need to be clarified/improved to increase readability and before we would recommend publication.

We thank the reviewer for their comprehensive comments on the study. Following these comments and in accordance with comments from other reviewers, we have made thorough revisions across all manuscript sections with an emphasis on clarifying and improving the readability of the text.

C1.1. General comments:

C1.1.1. My largest concern with the manuscript in its current form is that the relevance and implications of the research are unclear (the 'why' is missing). In the abstract is not indicated which scientific/societal question is exactly addressed by this paper and why this is needed to be answered. It mainly feels like an incremental research, that builds on previous studies by the same authors by expanding the time line and decomposition of the main drivers of change in planetary boundary transgression. But what are the implications of the results in this study? What does a regional decomposition mean for preventing further transgressions for example? I am not yet sure that the paper provides enough scientific advances to justify publication in a Nature journal, but I think the manuscript would benefit greatly from some more societal context, some elaboration on the why and discussion on implications.

We have made multiple revisions throughout the abstract, introduction and discussion sections to better address the relevance and implications of the study. Please find below summaries of the main revisions.

We have added more societal context when introducing the study, which is also key for better addressing the "why" of this study. In summary, societies depend on a stable Earth system, and human-driven freshwater change contributes to elevated risks of compromising this stability and freshwater security as such. Thus, we must better understand the extent, degree, and drivers of global-scale freshwater change that relates to these risks. We have revised how we contextualise our study, including L33–34, L53–55, and particularly L44–48 that read as follows:

"Cumulatively with other human-driven impacts on the Earth system, disruptions of the freshwater cycle thus contribute to a feedback loop; while societies depend on a stable Earth system, they create pressures that threaten its continued stability and then put themselves at an increasing risk. This interplay motivates a strong need to assess and understand freshwater change at the global scale."

We have discussed the implications of our results in more detail and addressed how our results go beyond an incremental update of the previous PB-FW analysis. We are confident that especially with the spatially and temporally detailed decomposition of climatic and direct human drivers on the manifold ways of freshwater change, our study is a useful approach to bridge the PB-FW (and the PBs framework in general) with regionally based insights that can help in addressing the underlying drivers of PB-FW transgressions. For instance, the outcome of our synthesising analysis (L326–332) shows how our approach can be used to identify particularly vulnerable regions in a globally uniform but regionally applicable way. These matters were largely left out of the scope of Porkka et al. (2024), due to their emphasis on conceptually and methodologically redefining the PB-FW – as well as the lack of suitable data. These revisions are best summarised by:

L27–31: *"Our coherent unpacking of the global PB-FW transgression into regional components is a notable advancement for analysing PBs across scales. In this work, we thus demonstrate the utility of the PB-FW for improved understanding of the extent, degree and drivers of global freshwater change, further guiding mitigation and adaptation strategies in response to it."*

L75–81: *"We then decompose the historical contributions of direct human forcing (DHF; including water use and land cover change, for instance) and climate-related forcing (CRF) to PB-FW metrics at global and regional scales, which advances from the previous PB-FW analysis by providing an in-depth outlook into the global and regional drivers of freshwater change. This demonstrates how the PB-FW can be used as a tool to study typologies of regions and configurations of freshwater change, which may contribute to freshwater-related risks in the Earth system, given historical dependencies and impacts on blue and green water resources."*

L356–358: *"We thus believe that our results contribute to bridging the scales between the PBs framework, which is largely focused on the broad Earth system, and regional-scale research, which may allow for more tangible points and means of action to practically address freshwater change."*

We have removed the "Concluding remarks" section and sharpened the end of our discussion section to better articulate what we consider as the main merits of this study and what we see as the ways forward. This summarising part of the discussion begins in L393–395 with:

"Improved understanding of global freshwater change and the interplay between climatic and direct human forcing that drive its regional features enhances the capacity to assess, mitigate, or possibly adapt to its adverse impacts on the Earth system and societies."

C1.1.2. I miss an underpinning of why the 'variance in the occurrence of local deviations under the baseline scenario' is set as the planetary boundary for green and blue freshwater? Could the authors indicate what it exactly means and why this value is considered a 'boundary'? Do I understand this correctly as 'the percentage of global area that is at the same time experiencing a drought or a flood'?

The justification for defining the global and regional reference boundaries (including the PB-FW) is based on the extended argumentation by Porkka et al. (2024). It indeed translates approximately to "the percentage of global area that is at the same time experiencing a drought or a flood." However, we choose not to call local deviations droughts or floods, as we focus on local streamflow or soil moisture anomalies quantified against a preindustrial baseline, being in line with the PBs framework. We have now clarified the reasoning why we suggest that variance in the occurrence of local deviations under the baseline scenario can serve as a reference boundary for green and blue water, both in the main text and in the methods section:

L92–95: *"Following Porkka et al.[16], we assume that widespread deviations beyond the baseline scenario variability elevate gradual or abrupt systemic risks related to freshwater change and thus use its upper end as a reference boundary to represent these risks. At the global scale, the upper end reference boundary corresponds to the PB-FW[16,18]."*

L551–558: *"Here, following Porkka et al.[16] and the PBs framework[18], we presume that widespread (regional or global) simultaneous occurrence of anomalous conditions beyond pre-industrial-like baseline conditions contributes to or may trigger abrupt or gradual systemic shifts, which, consequently, contribute to freshwater-related risks in the Earth system. We chose the upper end of baseline scenario variability as a precautionary reference boundary to represent these risks, since it demarcates conditions that occurred rarely (expectedly once in every 20 years) under water cycle conditions that were yet largely undisturbed by direct and indirect human pressures. At the global scale, the upper end of baseline variability for dry and wet local deviations corresponds to the PB-FW[16,18]."*

At the same time, we acknowledge that defining the local, regional, and global boundaries remains a presumption that may be challenged and adjusted with better knowledge on how freshwater change translates to unacceptable systemic risks in the Earth system. This is discussed in L384–392 as follows:

"Due to the absence of systematic studies to reflect local and regional variation in the levels of freshwater change that translate to unacceptable Earth system risks, both grid cell-specific local bounds (5th–95th percentile bounds in streamflow and soil moisture) and regionally aggregated thresholds (95th percentile of local deviation occurrence in the baseline scenario) are set in a globally uniform manner (Methods). Gathering systematic, empirically grounded evidence to evaluate these presumptions and possibly adjusting local, regional and global boundaries based on that evidence remains a priority for improving the conceptualisation and quantification of cascading impacts and Earth system risks due to freshwater change."

C1.1.3. It is not clear why some sort of hypothesis testing (at both global and regional level) is not carried out to identify whether the scenario contributions are significant, especially for DHF contributions which look quite similar to the baseline. Such tests seem to have been carried out for trend significance, but no information is provided for attribution.

We have now conducted hypothesis testing also for scenario contributions at both global and regional scales. Since we are interested in whether the difference in local deviation occurrence between two scenarios is significant but cannot assume that annual local deviation occurrences are normally distributed, we chose to use the paired Wilcoxon signed rank test, implemented in the R package "stats" (R Core Team, 2026). This test evaluates whether the median of the pairwise differences between annual values (in 1990–2019) of two scenarios is equal to zero (i.e. whether there is a systematic shift between the two scenarios) and therefore suits our purpose.

We have explained the use of the test in applicable instances in L172–175, L233–235, and in L601–603. Furthermore, we have made two additional modifications for the analysis:

1) Instead of how we originally derived regional scenario contributions from normalised local deviation occurrence, we now derive scenario contributions and test their significance based on non-normalised percentages of land area with local deviations, both globally and regionally. This change was done because normalised local deviation occurrence can be non-linear (according to Eq. 1–3). We have revised the respective parts in methods (Eq. 4–6; L606–610) and the exemplifying visualisation in Fig. 3 to reflect this change.

2) Adding hypothesis testing to scenario contributions allowed us to redesign Fig. 5. We have redrawn the figure and checked that its caption and corresponding text sections are in line with updates to the visualisation. We have also redrawn Fig. 6 and Supplementary Fig. 7 and updated Supplementary Table 1 following updates to Eq. 4–6, but changes to all were minor.

C1.1.4. Discussion regarding the regional trends and drivers and how they compare with existing regional studies can be improved. Linking this discussion to possible regional implications and recommendations for specific regional research would add value to the manuscript.

We have notably strengthened the discussion between regional trends and drivers and existing regional studies, which is associated with both Fig. 4 and Fig. 5. Related to Fig. 4, L208–218 now discuss how selected regional deviation occurrence patterns coincide with research of the same regions. Related to Fig. 5, L268–278 now discuss with regional studies how CRF and DHF can affect regional deviation occurrence in contrasting or temporally variable ways. To further discuss how our results could be used for regional implications and especially future regionally specific research, we have also revised parts of the section describing our synthesising analysis in L280–283 and L311–314, for instance.

While the data and methods used in our analysis are globally applicable and comparable, they are also notably uncertain. Thus, we see that the primary implication of our results for regional research would be to provide broad, globally comparable characterisations of freshwater change, which remain to be looked into more detail using data and methods tailored within each regional context. In the discussion section, we have summarised how we envision our analysis to be best used at the regional scale in L347–356:

"Our consequent exploratory analysis, which appears to suggest alignment between freshwater change and human pressures on ecosystems (Fig. 6), may additionally serve as an early-stage validation for our method in distinguishing particularly vulnerable regions. Therefore, our analysis can point to prospects for further studying freshwater change and vulnerabilities to its impacts in specific regions, along with a globally comparable characterisation of the freshwater change that each region is going through (e.g. blue vs. green water, dry vs. wet deviations). Regional implications made with plausible confidence, however, remain to require additional, case-dependent research and validation. Nevertheless, our regional results are directly compatible with the PB-FW, showing the high degree of spatial granularity underlying its global transgression."

C1.2. Specific comments:

C1.2.1. L14: The first line of abstract seems to be at odds with L325-327.

We have revised selected instances to improve the discussion on how increasing frequencies of anomalous streamflow and soil moisture conditions (our analysis) and elevated Earth system risks (our goal to understand) are linked, such as in L92–95 in response to comment C1.1.2. We have correspondingly revised the first sentence of the abstract, and L17–18 now read as:

"Human-driven freshwater change relates to elevated Earth system risks, which motivates analysis to better understand its global characteristics."

C1.2.2. L102: Definition of persistently transgressed is not clear. Looks like this comes from Porkka et al. (2024), so could be a good idea to reintroduce in this manuscript. Also, some explanation on why this persistent transgression occurs after 1940 would be beneficial to the reader.

We have removed the phrase "persistent" from the manuscript to avoid confusion with Porkka et al. (2024). In their case, "persistent" referred to a year after which the moving (trailing) 10-year mean of land area with local deviations (for a region) did not return below the upper end of pre-industrial variability. We did not conduct a similar analysis here and thus prefer not repeating the same phrase. L114–115 now read as:

"The global occurrence of local deviations transgressed the PB-FW at around the 1940s (Fig. 1), which demonstrates long-standing alterations in the water cycle."

C1.2.3. L106: The phrase "indicates … elevated freshwater related risks in the earth system" may be better suited for discussion section.

We have moved this phrase to the discussion section. L335–337 now read as:

"Our findings highlight the continued increase in PB-FW transgression over the early 21st century (Fig. 1), which indicates elevated freshwater-related risks in the Earth system."

C1.2.4. Fig.2: Plotting the upper end of variability would be useful as this is also used to define the upper limit.

We have now plotted also the upper end of baseline variability in Fig. 2, and added a sentence to point to it in L141–142:

"In the case of dry deviations, local deviation occurrence shows more than a doubling even when compared to the upper end of baseline variability (Fig. 2c–d)."

C1.2.5. L161: It is not clear why hydroBASINS level 4 is chosen in particular for regional analysis

We chose to use HydroBASINS level 4 as our regional division because it provides a hydrologically viable intermediate level of detail, which can be adequately used to aggregate land areas of 0.5-degree grid cells that can cover up to ~3,000 km^2 in physical land area (when at the Equator). The selected HydroBASINS level 4 catchments have a median area of 60,000 km^2 and a mean area of 103,000 km^2. We consider that the average number of cells captured within a region would become insufficiently low, if we used a much more detailed regional division. HydroBASINS is also constructed hierarchically based on flow directions (Lehner and Grill, 2013), which allows distinguishing between sub-catchments of large basins. We have now better justified the use of HydroBASINS level 4 both in the main text and in the methods section as follows:

L182–185: *"We used the HydroBASINS level 4 catchment delineation[37] to assess these trajectories and to disaggregate the global PB-FW transgression into regional components. HydroBASINS level 4 is a hydrologically viable and sufficiently detailed zoning, which additionally allows for distinguishing between sub-catchments of larger basins (Methods)."*

L569–575: *"We estimated the regional occurrence of local deviations within HydroBASINS level 4 catchment boundaries[37], which are hierarchically nested sub-catchments delineated based on flow directions. The hydrological viability and the consistently detailed intermediate resolution made it a good choice for our analysis. Out of total 1,341 catchments in the original HydroBASINS level 4 polygon layer, 41 had more than 50% permanent ice cover and 20 did not overlap with hydrological data from ISIMIP. Thus, the final count of analysed regions was 1,280, with mean area of 103,000 km^2 and median area 60,000 km^2."*

C1.2.6. Fig. 3 and methods: It is not clear why 1990-2019 is the period chosen for regional analysis

We set the regional analysis period to cover years 1990–2019 due to two reasons: 1) we wanted to be as close to the present day as possible (being constrained to 2019 by data availability, however), and 2) we also wanted to decrease the impact of interannual variability (stemming from e.g. short-term climate variability) in the regionally aggregated local deviation occurrence. This is why we averaged local deviation occurrence over 1990–2019 and derived scenario contributions using the same time period. We have added reasoning for taking 30-year means in the captions of Fig. 3–6 and in L592–594 and L597–601 in the methods section.

C1.2.7. L188-190: These lines are difficult to follow

Our intention with these lines was to argue that the (low) degree of regional deviation occurrence might not directly indicate the degree of hydrological alteration. Along with revisions to strengthen the discussion between our findings and existing regional studies (C1.1.4), we have rephrased these lines and L212–218 now read as:

"Additionally, while some regions e.g. in Central Europe and western North America for dry streamflow and soil moisture deviations are yet to transgress their region-specific upper end boundaries, simply deviation occurrence does not fully capture how hydrological change is or is not occurring in the region. This is because CRF and DHF can act counterproductively, and notable human impacts on regional hydrology, such as large-scale damming in Europe[40] or forest cover loss in North America[41], may not turn out visible in these maps that only display the net outcome."

C1.2.8. Synthesis: It is not clear why the median of four percentiles is used to compare relative contribution. Why not aggregate the (area of) deviations first and then rank the percentiles to assess the overall contribution?

Our phrasing might have been misunderstood here; actually, we did first aggregate local deviations regionally, and then ranked the percentiles, separately for four metrics (dry streamflow deviations, wet streamflow deviations, dry soil moisture deviations, wet soil moisture deviations). Our intention with the synthesis was to create four different metrics depicting different types of change in blue and green water and find regions where all four metrics represent a high degree of change in a scenario (when comparing globally). This is why we used the median of the four ranks to yield the overall scenario contribution. To clarify this, we have revised phrasing both in the main text and in the methods section (L619–625). In the main text, L280–291 now read as:

"To demonstrate how our approach can be used to identify particularly affected or vulnerable regions, we synthesised how CRF and DHF affect overall regional deviation occurrence by comparing their contributions in each of the four subcomponents consisting of dry and wet deviations for both streamflow and soil moisture. For each subcomponent, we ranked all regions using global percentile ranks of scenario contributions relative to interannual variability in the baseline scenario (Methods). Higher percentile ranks for one subcomponent in a region indicate greater contribution of CRF or DHF to increasing deviation occurrence in that subcomponent, compared to other regions. When taking the median of four independent percentile ranks across the four subcomponents – each representing a different facet of the freshwater cycle – higher median percentile ranks signal greater deviation across many subcomponents and thus greater overall contribution of CRF or DHF on pervasive systemic freshwater changes (Fig. 6). In other words, this ranking shows where climatic or direct human drivers are the most prominent across blue and green water."

C1.2.9. L282-286: Since this paragraph is mostly "exploratory", as in it intends to visually identify some association between scenario contributions and the three auxiliary variables (presumably to guide further research), it might be a good idea to also qualify these sentences with words such as "likely", "appears to", etc. (also for related parts in discussion and conclusion)

We have rephrased the paragraphs outlining the results of our exploratory synthesis (L311–332) and the related parts in discussion (L347–350) to use more cautious language in terms of how certain or robust we perceive these findings.

C1.2.10. L357-360: It's not clear what exactly is meant by "…takes a step forward" and "offers an actionable use case". Do these relate to Fig. 6c-e?

Following revisions clarifying the relevance and implications of our study (in response to comment C1.1.1.), we have removed the concluding remarks section where these phrases were originally placed. However, what we meant with them is how we see our study as an advance in using the PB-FW for better understanding freshwater change in the Earth system, which can further act as a link across scales to better connect global and regional insights and realities of how freshwater resources are changing. We have now discussed this ambition e.g. in L356–358, and in the end of the discussion section in L401–408 that read as:

"The external, non-biophysical drivers of that dependence (e.g. trade markets[63]) may, however, be located far from the biophysical change location, which complicates how human decisions ultimately affecting local and regional water resources are made[64,65]. Therefore, coupling these initial driving forces to the pathways ending in freshwater-related Earth system risks would be one of the most important next steps. Advancing this line of research could weave together the PB framework's biophysically oriented global scale thinking with more holistic and societally actionable deliberation of the drivers and impacts of freshwater change in the Earth system."

C1.2.11. L381: It may be beneficial for the readers to note that the baseline conditions in this manuscript also differ from Porkka et al (2024) where pre-industrial simulations are used to define the baseline as opposed to counterfactual simulations here.

The reason behind this difference lies in how we use simulations from two different ISIMIP simulation rounds. However, the common denominator between the baseline scenarios here and in Porkka et al. (2024) is that human pressures on the freshwater cycle are largely absent, as stated in the simulation protocol for the counterfactual ISIMIP 3a simulations used here (Frieler et al., 2024). We elaborate on differences in the selected model ensembles in the methods section (L490–499) and have added a more explicit remark about the difference in baseline conditions in L428–430 that now read as:

"These conditions are thus adequately representative of pre-industrial-like reference conditions desired for the planetary boundaries framework[18], although they slightly differ from the nominally pre-industrial reference conditions used by Porkka et al.[16] for the initial PB-FW analysis."

C1.2.12. L418: It's not clear why this mismatch occurs and why this justifies removing ensembles from analysis in the main text, especially since some initial years are discarded anyway owing to model spinup (L456).

To further investigate this mismatch, we have first checked that it is repeated in the original data downloaded from ISIMIP and is thus not a result of our own data processing. Furthermore, we have been in contact with one of the modellers performing ISIMIP 3a simulations to scope potential reasons for the issue. We found out that there can be variation in how input data for different direct human forcings (e.g. reservoirs or land use) are prepared prior to the simulations, particularly between the 1901soc (no major DHF) and histsoc (historical DHF) scenarios. In our case, this divergence shows as the mismatch between scenarios.

While this issue constrains our ensemble, we are unable to correct it since we cannot modify the input data and simulation setups of the GHMs participating in the ISIMIP 3a simulations. Given that true CRF and DHF at early 20th century levels should produce closely matching outcomes across all scenarios in our analysis (as is the case for most of our ensemble members), we consider that it is a more cautious choice to leave the mismatching ensemble members out of the main results. Without us having run these hydrological simulations ourselves, we are also unable to assess how widely the issue would potentially affect outputs during the later 20th and 21st century. To better justify removing some ensemble members from the main analysis, we have revised L476–481 that now read as:

"We assume that this behaviour stems from differences in scenario simulations' input data, because with CRF and DHF at early 20th century levels, all scenarios would be expected to be in near agreement with the baseline scenario. Given that we had no access to these input data, we decided to discard these ensemble members from the presented main results. However, global results (equivalent to Fig. 2) including also the discarded ensemble members are shown in Supplementary Fig. 12."

Additionally, to clarify the difference between the mismatch issue and traces of potential simulation spinups, we have explained how the spinup relates to hydrological stores within a model reaching an equilibrium only after having been run for a certain (variable, model-dependent) amount of time cycles. This is unrelated to how simulation input data between scenarios can be mismatching, as e.g. in Supplementary Fig. 10a–b, traces of spinup are visible without a systematic shift between the baseline and historical scenarios. L512–515 now read as:

"To avoid potential traces of GHM simulation spinup (i.e. time cycles required to run a model before hydrological stores reach an equilibrium) affecting the local bounds (exemplified e.g. in Supplementary Fig. 10a–b), we discarded years 1901–1910 from determining the local bounds and from all subsequent steps."

C1.2.13. L484: It is not clear why monthly means is chosen to estimate annual deviation and what its implications are.

We used annual means of monthly percentages of land area with local deviations to put more emphasis on regions that are experiencing more frequent local deviations within individual years. Should local deviations (at the grid cell scale) occur within a region during multiple months in a year, the monthly percentages of land area with local deviations will correspondingly be elevated during those months. This will further be reflected in the annual mean of monthly percentages. Taking annual means of monthly percentages also ensures consistency and comparability with Porkka et al. (2024). We have added a brief explanation of this in L544–546 that now read as:

"Taking annual means emphasises temporally extensive changes in streamflow and soil moisture since annual mean values are higher for years during which local deviations occur over multiple months."

References

Porkka, M., Virkki, V., Wang-Erlandsson, L., Gerten, D., Gleeson, T., Mohan, C., Fetzer, I., Jaramillo, F., Staal, A., Te Wierik, S., Tobian, A., Van Der Ent, R., Döll, P., Flörke, M., Gosling, S.N., Hanasaki, N., Satoh, Y., Müller Schmied, H., Wanders, N., Famiglietti, J.S., Rockström, J., Kummu, M., 2024. Notable shifts beyond pre-industrial streamflow and soil moisture conditions transgress the planetary boundary for freshwater change. Nat Water 2, 262–273. https://doi.org/10.1038/s44221-024-00208-7

Response references

Frieler, K., Volkholz, J., Lange, S., Schewe, J., Mengel, M., del Rocío Rivas López, M., Otto, C., Reyer, C.P.O., Karger, D.N., Malle, J.T., Treu, S., Menz, C., Blanchard, J.L., Harrison, C.S., Petrik, C.M., Eddy, T.D., Ortega-Cisneros, K., Novaglio, C., Rousseau, Y., Watson, R.A., Stock, C., Liu, X., Heneghan, R., Tittensor, D., Maury, O., Büchner, M., Vogt, T., Wang, T., Sun, F., Sauer, I.J., Koch, J., Vanderkelen, I., Jägermeyr, J., Müller, C., Rabin, S., Klar, J., Vega del Valle, I.D., Lasslop, G., Chadburn, S., Burke, E., Gallego-Sala, A., Smith, N., Chang, J., Hantson, S., Burton, C., Gädeke, A., Li, F., Gosling, S.N., Müller Schmied, H., Hattermann, F., Wang, J., Yao, F., Hickler, T., Marcé, R., Pierson, D., Thiery, W., Mercado-Bettín, D., Ladwig, R., Ayala-Zamora, A.I., Forrest, M., Bechtold, M., 2024. Scenario setup and forcing data for impact model evaluation and impact attribution within the third round of the Inter-Sectoral Model Intercomparison Project (ISIMIP3a). Geoscientific Model Development 17, 1–51. https://doi.org/10.5194/gmd-17-1-2024

Lehner, B., Grill, G., 2013. Global river hydrography and network routing: baseline data and new approaches to study the world's large river systems. Hydrological Processes 27, 2171–2186. https://doi.org/10.1002/hyp.9740

Porkka, M., Virkki, V., Wang-Erlandsson, L., Gerten, D., Gleeson, T., Mohan, C., Fetzer, I., Jaramillo, F., Staal, A., te Wierik, S., Tobian, A., van der Ent, R., Döll, P., Flörke, M., Gosling, S.N., Hanasaki, N., Satoh, Y., Müller Schmied, H., Wanders, N., Famiglietti, J.S., Rockström, J., Kummu, M., 2024. Notable shifts beyond pre-industrial streamflow and soil moisture conditions transgress the planetary boundary for freshwater change. Nat Water 2, 262–273. https://doi.org/10.1038/s44221-024-00208-7

R Core Team (2026). R: A Language and Environment for Statistical Computing. Vienna, Austria: R Foundation for Statistical Computing. Available at: https://www.R-project.org

Reviewer #2 (Remarks to the Author):

I co-reviewed this manuscript with one of the reviewers who provided the listed reports. This is part of the Nature Communications initiative to facilitate training in peer review and to provide appropriate recognition for Early Career Researchers who co-review manuscripts.

We thank the reviewer for their participation in co-reviewing the manuscript with another reviewer and their contributions to the associated comments.

Reviewer #3 (Remarks to the Author):

Review of "Regionally divergent drivers behind transgressions of the freshwater change planetary boundary" by Virkki et al.

Recommendation: Major revisions

This study examines the planetary boundaries of freshwater change. It uses the latest version of the corresponding ISIMIP simulations. Understanding the drivers of transgressing planetary boundaries is an important and timely topic and this study makes a worthwhile contribution to the literature. The manuscript is well written but I have several comments which need to be addressed. One particular weakness is that in my opinion the analysis methods are not described in any kind of detail.

We thank the reviewer for their detailed comments especially on the methodology of the study. We have now considerably revised and improved on how we report our methods and hope that it is now easier to assess them.

C3.1. Line 30: Volatility can potentially also contribute to this. Extreme precip following a dry period, water might run off on the surface instead of sinking into ground. E.g.

Nordling, K., Fahrenbach, N. L. S., and Samset, B. H.: Climate variability can outweigh the influence of climate mean changes for extreme precipitation under global warming, Atmos. Chem. Phys., 25, 1659–1684, https://doi.org/10.5194/acp-25-1659-2025, 2025.

Franzke, C., 2022: Changing temporal volatility of precipitation extremes due to global warming. Int. J. Climatol., 42, 8971-8983.

We have revised our introduction to outline that climate variability – which we consider capturing also volatility – is another form of anomalous hydrological conditions linking to human modifications of the freshwater cycle. Citing Nordling et al. (2025) as reference number 5, L34–39 now read as:

"Human activities, however, continue to modify the freshwater cycle, by direct water withdrawals and flow regulation, and through anthropogenic climate and land cover change, for instance[3–7]*. As freshwater is deeply connected to terrestrial and aquatic ecological processes, anomalous hydrological conditions – including increased frequency, magnitude, and variability of drying or wetting across water cycle elements – can destabilise ecosystems where freshwater availability governs their resilience*[8–10]*."*

C3.2. Line 71: Why this particular range? Extreme water scarcity can occur when water reservoir levels drop to about 13% of capacity.; e.g Cape Town in 2018 which then also coined the term Day Zero Drought.

We have now more prominently cited Porkka et al. (2024) who established the new PB-FW methodology (including the local variability range) with their extended argumentation, both in the main text (L83–86, L92–95) and in the methods section (L509–512). However, we also acknowledge that defining the local, regional, and global boundaries remains a presumption that may be challenged and adjusted with better knowledge on how freshwater change translates to unacceptable systemic risks. This is discussed in L384–392 as follows:

"Due to the absence of systematic studies to reflect local and regional variation in the levels of freshwater change that translate to unacceptable Earth system risks, both grid cell-specific local bounds (5th–95th percentile bounds in streamflow and soil moisture) and regionally aggregated thresholds (95th percentile of local deviation occurrence in the baseline scenario) are set in a globally uniform manner (Methods). Gathering systematic, empirically grounded evidence to evaluate these presumptions and possibly adjusting local, regional and global boundaries based on that evidence remains a priority for improving the conceptualisation and quantification of cascading impacts and Earth system risks due to freshwater change.

We would finally like to clarify that we define local variability bounds by percentiles, instead of percentages. According to the City of Cape Town (2018), the dam storage capacity of 13% was very close to the minimum that had been experienced during the 20 years preceding 2018, which would correspond to a value near the 0th percentile of that period. Thus, in that example case, the 5th percentile of storage capacities would translate to a higher value than 13%, signifying the difference between percentiles and percentages.

C3.3. Line 80: "Maximally large": It is disputable whether your ensemble is maximally large. You could perform your own additional simulations.

We have revised this sentence to use a less disputable phrasing, and L96–97 now read as:

"We employed a large ensemble of state-of-the-art global hydrological model (GHM) outputs from the Inter-Sectoral Impact Model Intercomparison Project (ISIMIP) simulation round 3a[33,34]."

C3.4. Line 105: Are those trends statistically significant? Do you compute linear trends? Do you take account of serial correlations? In Fig. 1 it is visible that the mean and median are different most of the time. This suggests that the data are non-Gaussian. Has this been taken account of in the trend analysis? More information is needed here.

We have revisited the technical implementation of our trend analysis and improved on reporting it. To assess trends, we now use the Theil-Sen slope estimator as implemented in the R package "zyp" (Bronaugh and Schoeneberg, 2023). The Theil-Sen slope is a linear slope estimate, which is commonly considered as robust against non-Gaussian data. In the implementation of the "zyp" package, statistical significance of the Theil-Sen slope is estimated by the Kendall test (Bronaugh and Schoeneberg, 2023). We have now reported the trend detection method in more detail when discussing related results for the first time in L116–119, as well as in the methods section in L565–567 that now read as:

"Growth rates for global deviation occurrence were estimated by linear Theil-Sen regression slopes, significance of which was tested with the Kendall test (two-sided test, significance level $p = 0.05$), using the R package zyp[35]."

Regarding serial correlations, our investigation suggests that they do not have a major impact on our global-scale trend estimates. In Table R3.1, we compare Theil-Sen slopes reported in the main text against linear trends estimated by ordinary least squares (OLS) on the same data. We also tested for normality of residuals from the Theil-Sen trend fits using the Shapiro-Wilk test (Table R3.1). As the Theil-Sen and OLS linear trend estimates are in a fair agreement, and residuals of Theil-Sen fits appear normally distributed in all cases except for one, we would suggest that serial correlations or non-Gaussian data do not have a major impact on our trend estimates. This is because we assume that the OLS estimates would be substantially different from Theil-Sen, and the residuals of the Theil-Sen fits would be more widely non-normal, if the underlying data was heavily affected by serial correlations or non-normality.

Table R3.1. Diagnostics for estimating the impact of serial correlations or non-normality in the reported Theil-Sen trend estimates. An asterisk (*) denotes test results that reject the Shapiro-Wilk test null hypothesis (values are normally distributed) at a significance level p = 0.05.

variable	deviation class	Theil-Sen slope reported in line	Reported slope	OLS trend slope	p-value for Shapiro-Wilk test of Theil-Sen fit residuals
streamflow	dry deviations	L143	0.12601	0.12390	0.91328
streamflow	dry and wet deviations	L118	0.18899	0.18255	0.15215
streamflow	wet deviations	L145	0.09103	0.07955	0.93721
soil moisture	dry deviations	L143	0.22987	0.21505	0.90324
soil moisture	dry and wet deviations	L118	0.23330	0.22601	0.04089 *
soil moisture	wet deviations	L145	0.08447	0.06694	0.20065

C3.5. I think the use of percentage points (pp) is somewhat non-standard. Why don't you provide the relative change in %?

In describing results, we use percentage points in contexts where we compare changes in our main analytical metric (land area with local deviations), which is expressed in percentages (of ice-free land area with local deviations). Absolute (percentage point) changes in this metric are relevant figures when reporting the results of the current analysis (L111–113) and when comparing our results to those of Porkka et al. (2024) (L123–125). However, we have attempted to clarify our phrasing in relevant instances and, if possible, provide both absolute (pp) and relative (%) changes.

C3.6. Line 243: Maybe you should discuss these regions in more detail.

We have made multiple revisions to strengthen the discussion between our findings and existing regional studies. For this instance, we have discussed the regions in question in more detail with associated references, and L274–278 now read as:

"Additionally, some regions are largely yet to transgress their region-specific upper end boundaries (Fig. 4), but the same regions may show comparatively strong CRF or DHF contributions (Fig. 5). This rapid and relatively recent freshwater change can be seen, for instance, in southwestern North America that has experienced recent climate-related droughts[45] and southern China that is a region with highly growing demands for blue and green water use[46]."

C3.7. Figure 6: This figure is hard to understand. In the legend you have the dark orange and blue colors but not the light colors. In the caption you talk about "shaded". I assume you mean the light colors. Maybe you should remove them if they play negligible roles.

We have removed the light-coloured box plots from Fig. 6c–e. Originally, box plots drawn using those colours visualised distributions of values (population, HANPP, MSA) in groups that consisted of a low number of regions (Supplementary Table 1). We consider that visualising distributions based on such small group sizes is not robust and therefore we have now removed boxes representing those groups.

C3.8. Lines 278-280: This sentence needs more explanation and discussion.

We have revised and expanded the sentence to allow for a more expressive interpretation. L321–325 now read as:

"High HANPP seems positively associated with increasing DHF contributions and negatively associated with increasing CRF contributions (Fig. 6d). As high HANPP corresponds to a high level of human-driven alteration (e.g. by land cover change) in ecosystems' carbon and energy flows[48], these associations may signal how the strongest human pressures on the land surface affect both land and freshwater systems."

C3.9. Line 375: You need to explain the procedure. It is totally unclear what has been done.

We would like to clarify here that the CRF detrending procedure is done by ISIMIP for the input data of the ISIMIP 3a GHM simulations (see Section 2.2. and Section 3.1.3. in Frieler et al., 2024). Because the procedure is done for the ISIMIP simulations' input data, we have no control over it, and it affects the GHM simulations in a consistent way; simulations run with different GHMs use counterfactual climate input data that has gone through the same detrending procedure at ISIMIP's end. Here, however, we only use hydrological simulation outputs, without using or modifying their input data that is affected by the CRF detrending procedure. To clarify that the CRF detrending procedure is not conducted by us, we have rephrased and cited relevant references in L423–426 that now read as:

"The CRF detrending procedure, done by ISIMIP for the simulation rounds' input data, removes the global climate change trend from historical climate reanalysis data, but retains their internal variability, thus allowing the detrended climate data set to be used for climate change impact attribution[33,66]."

C3.10. Line 411-412: Is soil moisture data used also at monthly time scale?

Both soil moisture and streamflow data were used at a monthly time scale. We have rephrased the respective sentence, and L461–463 now read as:

"Both streamflow and soil moisture data were delivered at a monthly time resolution, with streamflow in units m^3/s, and soil moisture in units kg/m^2."

C3.11. Line 415: These are model data, why do they have missing/negative values? How does the data infilling affect the results? There are of course various ways you can do data infilling.

Here, we would like to emphasise that we take hydrological model simulation outputs as they are provided in the ISIMIP repository by the different modelling groups conducting the simulations. Since we do not conduct the simulations ourselves, we are unable to investigate why missing or negative values are originally present in the model outputs.

However, to assess how largely this might affect our results, we checked how prevalent the missing or negative values are in the model outputs taken from ISIMIP. We found that for soil moisture, outputs from MIROC-INTEG-LAND contained up to 380 grid cells (out of total 67,420 grid cells) with up to 6,334 negative monthly values (in all global monthly values from years 1901–2019), depending on month, scenario and climate forcing data set. Additionally, in all scenarios, outputs from JULES-W2 forced with GSWP3-W5E5 were missing all streamflow data for the month of December in the year 2019. Any other ensemble members did not have missing or negative monthly streamflow or soil moisture values, thus restricting the infilling to be applied for 1–3 ensemble members only.

In the absence of more knowledge, such as hydrological observations, which would be specific to the grid cells in which infilling applies, we chose to adopt a simple and uniform infilling method based on the mean of non-missing and non-negative values. Given that the total counts of values to be infilled were zero for nearly all ensemble members and very low for those that needed infilling, we assume that the infilling has a negligible impact on our results. To clarify this, we have revised L465–470 that now read as:

"For 1–3 ensemble members, the data, when gathered from ISIMIP, contained sporadic instances of missing or negative monthly values, which were filled with the mean of all non-missing and non-negative values of the respective grid cell, month, and simulation scenario. Since up to 380 grid cells (out of 67,420) needed infilling for soil moisture, and the only case of missing data was found for an individual month of streamflow data, we assume that the infilling has a negligible impact on our results."

C3.12. Line 461-463: Does this differ from Time of Emergence estimations?

E.g. Hawkins, E., and R. Sutton (2012), Time of emergence of climate signals, Geophys. Res. Lett., 39, L01702, doi:10.1029/2011GL050087.

This description (now at L518–521) outlines how we mark local deviations occurring in a grid cell. When monthly streamflow or soil moisture values are outside of the local variability bounds, we mark the respective grid cell and a month as a local deviation, but we do not further specify the local deviation by how far beyond the local bounds the monthly streamflow or soil moisture value was.

We are thus unsure how the referred instance relates to Time of Emergence estimations of Hawkins and Sutton (2012), which we understand relating to when change in a given climate variable (signal) becomes emergent or significant in the midst of natural variability (noise).

C3.13. Line 465-466: Most drought studies use running means to look at anomalous conditions. For computing SPI/SPEI you typically use a 3-month or up to 36/38 month running mean. Is this been done here too? If not, why not?

When assessing monthly percentages of land areas with local deviations, as described in the referred instance (now at L523–526), we do not use running means. This is because at this stage of the analysis, we sum the physical land areas of grid cells marked as local deviations and relate the sum to total global or regional land area, yielding monthly percentages. However, we use temporal averaging in three specific instances:

1) When estimating annual occurrence of local deviations (globally or regionally), we take annual (calendar year) means of monthly land areas with local deviations, as described in L541–546.

2) When assessing global trajectories of local deviation occurrence based on annual values (Fig. 1–2), we plot running (trailing) 10-year means of the annual time series of percentages of land area with local deviations.

3) When presenting regional occurrences of local deviations (Fig. 4) and assessing scenario contributions (Fig. 5), we take 30-year means of local deviation occurrence over 1990–2019 (L597–601).

With instance 1), we aim to add weight on years where local deviations occur during many months compared to years with fewer monthly local deviations, and with instances 2) and 3), we aim to smooth the possible impact of interannual (i.e. short-term) variability on our aggregated results. We consider that these aims are largely parallel to how running means are typically applied in different drought studies.

C3.14. Eq. (1): To me the equation seems to provide negative values. You say it should be between 0 and 1.

We have rephrased the phrasing related to explaining how Eq. 1–3 are constructed and used, and L578–583 now read as:

"To compare the occurrence of local deviations across differently sized regions with variable ambient CRF and DHF, we normalised the regional land area with local deviations with respect to the median and upper end of baseline variability (Fig. 3a), according to Eq. 1–3. The normalised local deviation occurrence yields values in the interval [–1, ∞[, and particularly value zero when local deviation occurrence corresponds to the region-specific median of baseline variability and value one when local deviation occurrence is at the region-specific upper end of baseline variability."

C3.15. Line 525: You could also look at nonlinear contributions following study by:

Ma, Q. and C. Franzke, 2021: The role of transient eddies and diabatic heating in the maintenance of European heat waves: a nonlinear stationary wave perspective. Climate Dyn., 56, 2983-3002.

We have considered this suggestion but have decided to remain using the current method of simplistically comparing means of regional deviation occurrence yielded from different scenarios. We consider that adding a more advanced method to assess scenario contributions might complicate the manuscript with extended methodological descriptions and justifications in an unreasonably complex way and risk detracting it from its main narrative. We believe that our method, given methodological clarifications outlined above, is still adequate and feasible with respect to our study scope.

C3.16. Why do you use two different versions of the HYDE data base? Does this affect your results?

We have now updated our analysis to use the latest release of the HYDE database (version 3.5; Klein Goldewijk, 2025). Originally, we used HYDE 3.2.1 to create a mask depicting grid cells that are covered by permanent ice and thus omitted when calculating global ice-free land area with local deviations. HYDE 3.4 was used as auxiliary data on population counts in our synthesising analysis on the overall contributions of CRF and DHF on regional deviation occurrence. HYDE 3.4 data affected no subsequent results or analysis steps but was only used in visualisations presented in Fig. 6 and Supplementary Fig. 8.

All methodological updates combined (e.g. in response to C3.4.), did not affect our results besides minor instances (e.g. in L142–145). Regarding HYDE in particular, we have now updated the related version information in L533–534 and L632–634. Additionally, we have redrawn all figures and checked all provided numeric estimates in the manuscript to verify that they correspond with our data analysis following all methodological revisions.

Response references

Bronaugh, D., Schoeneberg, A., 2023. zyp: Zhang + Yue-Pilon Trends Package. Version 0.11-1. https://cran.r-project.org/web/packages/zyp/index.html

City of Cape Town, 2018. Water Outlook 2018 Report. Available at: https://resource.capetown.gov.za/documentcentre/Documents/City%20research%20reports%20and%20review/Water%20Outlook%202018%20-%20Summary.pdf

Frieler, K., Volkholz, J., Lange, S., Schewe, J., Mengel, M., del Rocío Rivas López, M., Otto, C., Reyer, C.P.O., Karger, D.N., Malle, J.T., Treu, S., Menz, C., Blanchard, J.L., Harrison, C.S., Petrik, C.M., Eddy, T.D., Ortega-Cisneros, K., Novaglio, C., Rousseau, Y., Watson, R.A., Stock, C., Liu, X., Heneghan, R., Tittensor, D., Maury, O., Büchner, M., Vogt, T., Wang, T., Sun, F., Sauer, I.J., Koch, J., Vanderkelen, I., Jägermeyr, J., Müller, C., Rabin, S., Klar, J., Vega del Valle, I.D., Lasslop, G., Chadburn, S., Burke, E., Gallego-Sala, A., Smith, N., Chang, J., Hantson, S., Burton, C., Gädeke, A., Li, F., Gosling, S.N., Müller Schmied, H., Hattermann, F., Wang, J., Yao, F., Hickler, T., Marcé, R., Pierson, D., Thiery, W., Mercado-Bettín, D., Ladwig, R., Ayala-Zamora, A.I., Forrest, M., Bechtold, M., 2024. Scenario setup and forcing data for impact model evaluation and impact attribution within the third round of the Inter-Sectoral Model Intercomparison Project (ISIMIP3a). Geoscientific Model Development 17, 1–51. https://doi.org/10.5194/gmd-17-1-2024

Hawkins, E., Sutton, R., 2012. Time of emergence of climate signals. Geophysical Research Letters 39. https://doi.org/10.1029/2011GL050087

Klein Goldewijk, K., 2025. HYDE data for the Global Carbon Budget (2025). https://doi.org/10.24416/UU01-F45D44

Nordling, K., Fahrenbach, N.L.S., Samset, B.H., 2025. Climate variability can outweigh the influence of climate mean changes for extreme precipitation under global warming. Atmospheric Chemistry and Physics 25, 1659–1684. https://doi.org/10.5194/acp-25-1659-2025

Porkka, M., Virkki, V., Wang-Erlandsson, L., Gerten, D., Gleeson, T., Mohan, C., Fetzer, I., Jaramillo, F., Staal, A., te Wierik, S., Tobian, A., van der Ent, R., Döll, P., Flörke, M., Gosling, S.N., Hanasaki, N., Satoh, Y., Müller Schmied, H., Wanders, N., Famiglietti, J.S., Rockström, J., Kummu, M., 2024. Notable shifts beyond pre-industrial streamflow and soil moisture conditions transgress the planetary boundary for freshwater change. Nat Water 2, 262–273.

Reviewer #4 (Remarks to the Author):

This manuscript provides a very interesting and relevant addition to the recent literature on the freshwater planetary boundary. Building on previous work by Porkka et al., the assessment of the PB-FW is extended to the last decades and deepened, particularly by providing regional attributions of climate change and direct human impacts on the two dimensions of the PB (green and blue water). The study is well-designed and the manuscript reads very well. I do have some suggestions and requests.

We thank the reviewer for their encouraging comments on the study. We agree with the suggestions to improve on how we discuss our findings and limitations and have now revised the text to more clearly and diversely articulate these perspectives.

C4.1. On a conceptual level, the PBs are by definition a global aggregate. Although I agree that regional differentiation is an important and interesting exercise, possible spatial differences thresholds are not considered. That is, for example, for some regions a 99th percentile of historical variability in soil moisture might have adverse systemic effects and for other ones it would be the 95th percentile. Although I would not advocate for a revised definition of the PB-FW itself, it can, however, reasonably be argued that downscaling the PB under the assumption that the globally aggregated 95th percentiles apply also at more local scales leaves out important local detail. If the authors agree, it would be good to see a more explicit discussion of this limitation.

We have done two main clarifications to more explicitly acknowledge and discuss this limitation, which stems from how the new PB-FW was defined by Porkka et al. (2024). First, we now clarify in more detail that the reference boundary defined by the upper end of baseline variability (that is the PB-FW at the global scale, numerically placing between 12–13%) can be different between regions based on regional variability in the baseline scenario. This means that for some regions, the upper end of baseline variability can numerically place higher than the global 12–13%, and, vice versa, some regions might have a lower numerical value for this upper end reference boundary. We have elaborated on this in the methods section, where L539–541 now read as:

"Both reference boundaries were set at the global scale and for each region separately, allowing the regional reference boundaries to scale based on the variance of local deviation occurrence in the baseline scenario."

However, regional scalability of the reference boundaries does not preclude the fact that we do not yet have robust, locally or regionally specific knowledge on the percentiles of baseline variability at which adverse systemic effects may emerge. This is an inherent limitation of the PB-FW, being also discussed by Porkka et al. (2024), who opted to use the 95th percentile since it demarcates conditions that occurred relatively rarely (expectedly once in every 20 years) under baseline conditions that are largely absent of major human modifications of the freshwater cycle. By retaining the choice of the 95th percentile as a reference boundary in this study, we ensure direct comparability of our results with the results of Porkka et al. (2024) (L83–95, L501–506) and the continuity of consistent PB-FW estimates approaching the present day following updates to hydrological model ensembles.

As another key revision, we discuss this key limitation and acknowledge that defining the local, regional, and global boundaries remains a presumption that may be challenged and adjusted with better knowledge on how freshwater change translates to unacceptable systemic risks. We have revised L384–392 as follows:

"Due to the absence of systematic studies to reflect local and regional variation in the levels of freshwater change that translate to unacceptable Earth system risks, both grid cell-specific local bounds (5th–95th percentile bounds in streamflow and soil moisture) and regionally aggregated thresholds (95th percentile of local deviation occurrence in the baseline scenario) are set in a globally uniform manner (Methods). Gathering systematic, empirically grounded evidence to evaluate these presumptions and possibly adjusting local, regional and global boundaries based on that evidence remains a priority for improving the conceptualisation and quantification of cascading impacts and Earth system risks due to freshwater change."

C4.2. The planetary boundaries can, of course, never be completely disentangled. For example, land cover change affects the climate, but both processes can and should still be considered separately in the PB framework. Indeed, the authors find that, globally, transgressions of the freshwater boundary are predominantly attributed to climate change. This is discussed in the paragraph starting in line 303, where this result is presented as a strength, because it underscores the interconnectedness of the Earth System. But could there also be negative implications of this result for the PB framework? Wouldn't it possibly undermine the validity of the PB-FW as a separate PB if transgressions are already captured by a (transgressed) climate PB? Here again, I do not argue for a fundamental revision of this PB, but a more critical reflection seems in place.

We have extended and diversified the discussion on how our PB-FW analysis relates to the PBs framework in general and especially the climate change PB (PB-CC). It is correct that in addition to the various non-climatic causes contributing to PB-FW transgression (e.g. water and land use), much of the PB-FW transgression is currently conditional to PB-CC transgression. However, the PB-FW, as applied in this study, can elucidate the different types of freshwater change (blue vs. green water; dry vs. wet deviations) and demonstrate the high degree of regional granularity underlying the global PB-FW transgression. These abilities are lacking from the PB-CC.

Decomposing the PB-FW transgression is essential also for understanding its potential impacts, which can differ between the different components of the PB-FW, and especially from the impacts of PB-CC transgression. The impacts of PB-FW transgression may be more confined spatially and harder to distinguish by the PB-CC only (e.g. basin-scale freshwater-supported ecosystem collapse) and they can also feed back to further PB-CC transgression (e.g. by land carbon balance alteration). Thus, the PB-FW, compared to the PB-CC, offers more ways to understand freshwater-related risks in the Earth system, which is at the conceptual core of the PBs framework (Rockström et al., 2009). Indeed, the previous PB-FW definitions suffered from similar issues in representing the manifold impacts of freshwater change in the Earth system, which motivated the redefinition by Porkka et al. (2024). For more in-depth reflections and better articulation of these matters, we have now rewritten a paragraph in the discussion section, and L359–374 now read as:

"Interpreting the PBs framework in light of our findings emphasises the interconnectedness of Earth system processes represented by individual PBs[52,53]. Even in the hypothetical absence of DHF, CRF alone would have caused a notable PB-FW transgression (Fig. 2). Given that a separate PB for climate change (PB-CC) exists[18], PB-FW could be argued to be redundant because its transgression is highly conditional to PB-CC transgression. We, however, conversely argue that the PB-FW can distinguish factors that the PB-CC cannot; the PB-CC cannot separate between widely variable regional patterns of the climate change impact on blue and green water (Supplementary Fig. 5), and in some regions, non-climatic factors may be much stronger contributors to freshwater change (Fig. 5). Similarly, the first PB-FW approaches that only considered human blue water consumption[2,22] and their further applications[26,54,55] neglect most of these factors (and the systemic influence of climate) and thus remain limited in how they can be used to understand freshwater-related risks in the Earth system. Therefore, in accordance with previous studies[1,32], we strongly advocate for considering the freshwater cycle as a highly dynamic Earth system component instead of a controllable tap. Similar applications and appraisals of other PBs, as well, could aid in addressing critiques of the framework[56] and in broader goals of using the framework to inform sustainable environmental governance and Earth system stewardship[31,57,58].

C4.3. Lines 123-125: This can be established statistically rather than through simple eyeballing. Please provide a more objective way of reporting this important difference between dry and wet deviations.

We attempted to better quantify this claim by analysing variation in the annual occurrence of local deviations in 30-year moving windows across the late 20th and early 21st century. However, differences in variation (standard deviation) between the moving windows are small and partially inconsistent between blue and green water and wet and dry deviations, especially for 1970–1999 compared to other moving windows (Table R4.1). Therefore, we consider that an objective way of reporting this difference would become a needlessly technical argument and have decided to remove this claim from the manuscript.

Table R4.1. Standard deviation of the annual occurrence of local deviations in 30-year moving windows for dry and wet deviations.

variable	deviation class	standard deviation of annual values between			
		1960–1989	*1970–1999*	*1980–2009*	*1990–2019*
streamflow	dry deviations	1.42	0.90	1.25	1.31
streamflow	wet deviations	1.13	1.22	1.02	1.07
soil moisture	dry deviations	1.37	0.85	1.62	2.11
soil moisture	wet deviations	1.24	1.39	1.17	1.14

Minor points:

C4.4. 155: This is about the past, so the past tense seems more appropriate.

We have rephrased this instance to use past tense. L175–178 now read as:

“Temporally, the divergence between CRF and DHF scenarios began to notably widen around the 1970s for all other components except for dry streamflow deviations (Fig. 2d–f), which shows how the impact of climate change has become more pronounced in the freshwater cycle during the late 20th and early 21st century.”

C4.5. 292 and elsewhere: “The last 15 years” is not entirely accurate as the analysis ends in 2019.

We have revised related phrasings in, for instance, L64–65 and L335–337 to be more accurate.

Response references

Porkka, M., Virkki, V., Wang-Erlandsson, L., Gerten, D., Gleeson, T., Mohan, C., Fetzer, I., Jaramillo, F., Staal, A., te Wierik, S., Tobian, A., van der Ent, R., Döll, P., Flörke, M., Gosling, S.N., Hanasaki, N., Satoh, Y., Müller Schmied, H., Wanders, N., Famiglietti, J.S., Rockström, J., Kummu, M., 2024. Notable shifts beyond pre-industrial streamflow and soil moisture conditions transgress the planetary boundary for freshwater change. Nat Water 2, 262–273. https://doi.org/10.1038/s44221-024-00208-7

Rockström, J., Steffen, W., Noone, K., Persson, Å., Chapin, F.S.I., Lambin, E., Lenton, T., Scheffer, M., Folke, C., Schellnhuber, H.J., Nykvist, B., de Wit, C., Hughes, T., van der Leeuw, S., Rodhe, H., Sörlin, S., Snyder, P., Costanza, R., Svedin, U., Falkenmark, M., Karlberg, L., Corell, R., Fabry, V., Hansen, J., Walker, B., Liverman, D., Richardson, K., Crutzen, P., Foley, J., 2009. Planetary Boundaries: Exploring the Safe Operating Space for Humanity. Ecology and Society 14. https://doi.org/10.5751/ES-03180-140232

Point-by-point response to the reviewers’ comments of

Regionally divergent drivers behind transgressions of the freshwater change planetary boundary

Reviewer #1 (Remarks to the Author):

I would like to thank the authors for extensive answers to the reviewers comments. I can see the manuscript increased in clearity from answering to those comments.

My initial concerns, that are unrelated to the quality of the analysis and the writing, but more fundamental to the choice of these indicators to identify planetary boundaries for freshwater, remain.

Although the authors have now made the link to societal impact, and explained that the indicators can be used to support adaptation decisions, I still find these indicators a bit arbitrary. Argueing that the 'societies depend on a stable Earth system', seems to be a bit simple.

At the same time, I do not have a solution for this, as I think this issue is related to defining 'planetary boundaries' in general, also for the other elements of the planetary boundaries. Coming up with an indicator that is really meaningful and has clear implications when transgressed, is rather difficult. Still it is a very valuable framework to show the state of our Earth System and raise awareness of where it is heading.

We thank the Reviewer for the time and effort put into reviewing the manuscript again after our revisions. We are glad to hear that our responses were adequate, and that the manuscript has increased in clarity.

We agree that the fundamental problem of creating indicators that both align with the planetary boundaries (PBs) framework and are meaningful and relevant in practice remains challenging. While the underlying argument that societies depend on a stable Earth system is comparatively simple, it has been central to the PBs framework from its inception (Rockström et al., 2009) to its most recent update (Richardson et al., 2023). While we chose to follow the framework by using the PB for freshwater change (PB-FW) in this study, we considered a conceptual redevelopment of the PB-FW indicators themselves to be beyond the scope of this work, due to recently being discussed and done by Porkka et al. (2024).

Nevertheless, we recognise that the manuscript could more clearly acknowledge that the use of comparatively simplistic indicators also reflects broader conceptual limitations of the PBs framework. We have rephrased a paragraph in the Discussion to better articulate these limitations, and lines 326–338 now read as:

"Perhaps most importantly, however, linking indicators that depict frequencies of anomalous streamflow and soil moisture conditions with an elevated level of freshwater-related Earth system risks is a general presumption stemming from the PBs framework[16,17,18]. Due to the absence of systematic studies to reflect local and regional variation in the levels of freshwater change that translate to unacceptable Earth system risks, both grid cell-specific local bounds (5th–95th percentile bounds in streamflow and soil moisture) and regionally aggregated thresholds (95th percentile of local deviation occurrence in the baseline scenario) are set in a globally uniform manner (Methods). In general, it is notably difficult to devise globally applicable indicators with clear implications when transgressed, while also comprehensively representing highly complex Earth system processes, such as the freshwater cycle. Gathering systematic, empirically grounded evidence to evaluate these presumptions, and possibly adjusting local, regional and global boundaries based on that evidence remains a priority for improving the conceptualisation and quantification of cascading impacts and Earth system risks due to freshwater change."

Response references

Porkka, M., Virkki, V., Wang-Erlandsson, L., Gerten, D., Gleeson, T., Mohan, C., Fetzer, I., Jaramillo, F., Staal, A., te Wierik, S., Tobian, A., van der Ent, R., Döll, P., Flörke, M., Gosling, S.N., Hanasaki, N., Satoh, Y., Müller Schmied, H., Wanders, N., Famiglietti, J.S., Rockström, J., Kummu, M., 2024. Notable shifts beyond pre-industrial streamflow and soil moisture conditions transgress the planetary boundary for freshwater change. Nat Water 2, 262–273. https://doi.org/10.1038/s44221-024-00208-7

Richardson, K., Steffen, W., Lucht, W., Bendtsen, J., Cornell, S.E., Donges, J.F., Drüke, M., Fetzer, I., Bala, G., von Bloh, W., Feulner, G., Fiedler, S., Gerten, D., Gleeson, T., Hofmann, M., Huiskamp, W., Kummu, M., Mohan, C., Nogués-Bravo, D., Petri, S., Porkka, M., Rahmstorf, S., Schaphoff, S., Thonicke, K., Tobian, A., Virkki, V., Wang-Erlandsson, L., Weber, L., Rockström, J., 2023. Earth beyond six of nine planetary boundaries. Science Advances 9, eadh2458. https://doi.org/10.1126/sciadv.adh2458

Rockström, J., Steffen, W., Noone, K., Persson, Å., Chapin, F.S.I., Lambin, E., Lenton, T., Scheffer, M., Folke, C., Schellnhuber, H.J., Nykvist, B., de Wit, C., Hughes, T., van der Leeuw, S., Rodhe, H., Sörlin, S., Snyder, P., Costanza, R., Svedin, U., Falkenmark, M., Karlberg, L., Corell, R., Fabry, V., Hansen, J., Walker, B., Liverman, D., Richardson, K., Crutzen, P., Foley, J., 2009. Planetary Boundaries: Exploring the Safe Operating Space for Humanity. Ecology and Society 14. https://doi.org/10.5751/ES-03180-140232

Reviewer #2 (Remarks to the Author):

I co-reviewed this manuscript with one of the reviewers who provided the listed reports. This is part of the Nature Communications initiative to facilitate training in peer review and to provide appropriate recognition for Early Career Researchers who co-review manuscripts.

We thank the Reviewer for the time and effort put into reviewing the manuscript again after our revisions.

Reviewer #3 (Remarks to the Author):

The authors have addressed all my comments. I recommend to accept the manuscript for publication now.

We thank the reviewer for the time and effort put into reviewing the manuscript again after our revisions and are glad to hear the recommendation for publishing the manuscript.

Reviewer #4 (Remarks to the Author):

I thank the authors for their careful and detailed replies and revisions and am happy to recommend publication of this work.

We thank the Reviewer for the time and effort put into reviewing the manuscript again after our revisions and are glad to hear the recommendation for publishing the manuscript.
